# Oxidative-Stress-Related Genes in Osteoporosis: A Systematic Review

**DOI:** 10.3390/antiox12040915

**Published:** 2023-04-12

**Authors:** Guadalupe León-Reyes, Anna D. Argoty-Pantoja, Adriana Becerra-Cervera, Priscilla López-Montoya, Berenice Rivera-Paredez, Rafael Velázquez-Cruz

**Affiliations:** 1 Genomics of Bone Metabolism Laboratory, National Institute of Genomic Medicine (INMEGEN), Mexico City 14610, Mexico; abecerra@inmegen.edu.mx (A.B.-C.); priscilla_lopez92@comunidad.unam.mx (P.L.-M.); rvelazquez@inmegen.gob.mx (R.V.-C.); 2 Research Center in Policies, Population and Health, School of Medicine, National Autonomous University of Mexico (UNAM), Mexico City 04510, Mexico; argotyanna@comunidad.unam.mx (A.D.A.-P.); bereriverap@comunidad.unam.mx (B.R.-P.); 3 National Council of Science and Technology (CONACYT), Mexico City 03940, Mexico

**Keywords:** oxidative stress, osteoporosis, bone mineral density, gene, single nucleotide variants

## Abstract

Osteoporosis is characterized by a decline in bone mineral density (BMD) and increased fracture risk. Free radicals and antioxidant systems play a central role in bone remodeling. This study was conducted to illustrate the role of oxidative-stress-related genes in BMD and osteoporosis. A systematic review was performed following the PRISMA guidelines. The search was computed in PubMed, Web of Sciences, Scopus, EBSCO, and BVS from inception to November 1st, 2022. The risk of bias was evaluated using the Joanna Briggs Institute Critical Appraisal Checklist tool. A total of 427 potentially eligible articles exploring this search question were detected. After removing duplicates (n = 112) and excluding irrelevant manuscripts based on screenings of their titles and abstracts (n = 317), 19 articles were selected for full-text review. Finally, 14 original articles were included in this systematic review after we applied the exclusion and inclusion criteria. Data analyzed in this systematic review indicated that oxidative-stress-related genetic polymorphisms are associated with BMD at different skeletal sites in diverse populations, influencing the risk of osteoporosis or osteoporotic fracture. However, it is necessary to look deep into their association with bone metabolism to determine if the findings can be translated into the clinical management of osteoporosis and its progression.

## 1. Introduction

Bone is a metabolically active tissue that experiences continuous remodeling via two reciprocal processes: bone formation and bone resorption. This coordinated process is carried out by osteoclasts, osteoblasts, and osteocytes [1]. Disturbance in the activity of these specialized cells would ultimately lead to several diseases, such as osteoporosis [2]. Osteoporosis is a progressive bone disease characterized by a decrease in bone mineral density (BMD) that increases the fracture risk [3]. According to many studies, genetic and environmental factors contribute to the development of the disease by about 70% and 30%, respectively [4].

The activity of bone cells can be influenced by various cellular factors, including nutrients, endocrines, cytokines, growth factors, and free radicals [5]. Free radical products of oxygen metabolism are generated from the excessive production of reactive oxygen species (ROS) via the mitochondrial electron-transport chain (due to electron leak in the respiratory chain) by enzymatic complexes, such as xanthine oxidase and nicotinamide adenine dinucleotide phosphate (NADPH) oxidases, or under environmental stimuli (e.g., cytokines, ultraviolet radiation, drugs, pollution) [6]. 

The antioxidant system consists of agents such as vitamins C and E, or reduced glutathione (GSH), and antioxidant enzymes such as glutathione peroxidase (GPX), superoxide dismutase (SOD), and paraoxonase (PON) that neutralize ROS. When there is an increase in the production of ROS and a failure of the antioxidant systems to neutralize them, an oxidative stress state could occur [7]. Adverse effects of oxidative stress result from damage to cell structures due to lipid and protein oxidations. In addition, ROS could modify mitochondrial and nuclear DNA integrity by increasing the risk of mutations through the deregulation of redox-sensitive transcription factor activities [7]. When DNA repair mechanisms are overwhelmed, cells undergo apoptosis or necrosis, which can lead to tissue damage [8]. Therefore, ROS has been broadly associated with health complications, including cancer, neurological disorders, atherosclerosis, diabetes mellitus, and osteoporosis [9].

Accumulating evidence suggests that bone biology is mainly affected by redox balance regulation and that targeting ROS production in bone cells might be an essential approach to preventing bone damage [10]. Briefly, controlled production of free radicals by normally functioning osteoclasts could accelerate the destruction of calcified tissue and assist in bone remodeling [11]. Generated superoxide from osteoclasts directly contributes to bone degradation; however, osteoblasts produce antioxidants such as GPX to protect against ROS [12]. Therefore, oxidative stress has an active function on osteoblast activity and mineralization [12]. Under these conditions, the imbalance between ROS production and antioxidant mechanisms may negatively affect bone metabolism and contribute to osteoporosis [13]. Twin and family studies have shown that genetic variants could explain 50–80% of the risk of osteoporosis [14]. In this context, genetic association studies have been crucial in providing information about the genetic architecture of osteoporosis and bone fracture predisposition [15]. Because of the crucial role of oxidative stress in bone turnover, this systematic review summarizes the principal single nucleotide variants (SNVs) involved in oxidative stress genes associated with osteoporosis. With this information, in the future, we could propose new therapeutic approaches for osteoporosis treatment to improve patients’ quality of life.

## 2. Materials and Methods

### 2.1. Search Strategy and Eligibility Criteria

We followed the recommendations in the Preferred Reporting Items for Systematic Reviews and Meta-Analyses (PRISMA) guidelines [16]. This protocol was registered in PROSPERO (ID: CRD42023408104). Thus, we performed an extensive literature search using PubMed, Web of Science, Scopus, EBSCO, and BVS, mainly based on the search terms, with English language restrictions and independently applied inclusion and exclusion criteria to screen titles and abstracts of the remaining articles. There were no restrictions based on the race/ethnicity or gender of participants. The search strategy included the terms: “gene”; “single nucleotide polymorphism”; “genetic variant”; “oxidative stress”; “antioxidant”; “bone mineral density”; “osteoporosis”; and “fractures” (Table S1). These were seeded in text word searchers and the “related articles” function was used to broaden the search. We also reviewed publications cited in references using these search words for relevant studies that were not identified. In addition, all searches were conducted with no period time specified. 

### 2.2. Study Selection

We searched all abstracts for potentially relevant publications. Studies meeting the following criteria were included: (1) original studies evaluating the relationship between different oxidative status genes in bone metabolism; (2) original studies conducted in the adult population (aged ≥ 18 years); (3) articles written in English; (4) studies reported as an original research paper in a peer-reviewed journal; (5) studies adequately describing their samples (e.g., diagnostic criteria, and source of samples), and methods such that the experiments could be replicated; (6) full text of the selected articles available for retrieval.

Papers were excluded if (1) the study enrolled children, cell cultures, or animals; (2) the study’s full-text version was written in a language other than English; (3) the articles were reviews, letters to the editor, case reports, or abstracts presented at scientific events; (4) the full-text version of the article was unavailable; and (5) the studies did not report sufficient information on the role of genes encoding enzymes involved in regulating oxidative stress or on oxidative stress levels. Concordance was evaluated through Fleiss’ kappa statistic. Any disagreement between the investigators was resolved by consultation with the senior coordinators of the project (G.L.-R. and R.V.-C.), allowing for the final selection of the papers to be included in this systematic review.

### 2.3. Data Collection and Analysis

#### 2.3.1. Data Extraction and Management

Data extraction was performed independently by three researchers (P.L.-M., A.B.-C., and B.R.-P.) and validated by independent researchers (A.D.A.-P. and G.L.-R.). The data were added to a predetermined and standardized data form using Microsoft Excel 365. Disagreements between researchers were discussed and resolved.

#### 2.3.2. Risk of Bias

The risk of bias of selected publications was evaluated using the Joanna Briggs Institute (JBI) Critical Appraisal Checklist, with a score of ≥5, 4, and <4 indicating low, moderate, and high risk of bias, respectively [17]. Two researchers (P.L.-M. and A.B.-C.) independently performed the risk of bias evaluation, and a third researcher (A.D.A.-P.) defined disagreements.

#### 2.3.3. Data Synthesis

The data synthesis describes the SNVs found grouped into gene families, the outcomes with their measurement method, and the observed association. Due to the variability between studies and the need for more detailed information (SNVs, outcomes, methods used to assess the variables of interest, data management, etc.), meta-analysis could not be performed. However, the data extracted from each included study allowed us to describe the variables of interest.

## 3. Results

### 3.1. Systematic Research

**Figure 1:**
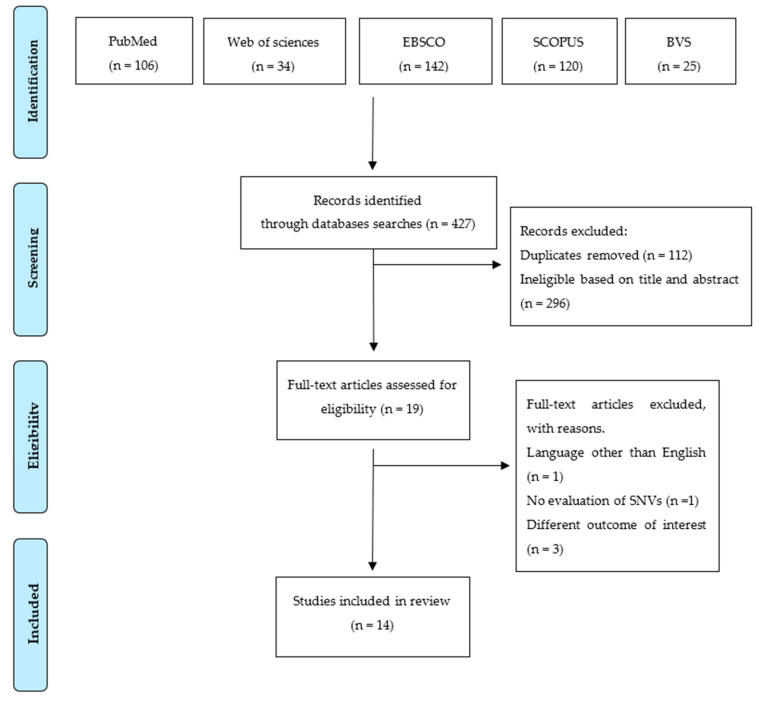
PRISMA 2020 flow chart describing the screening process.

The flow diagram of the literature search process is reported in Figure 1. We detected a total of 427 relevant articles, and we removed 112 duplicates. Two hundred ninety-six articles were excluded based on the evaluation of titles and abstracts for not corresponding to the aim of this review. The excluded articles were conducted in Australia (1), Austria (6), Belgium (3), Brazil (8), Canada (5), Chile (1), China (94), Czech Republic (1), Denmark (1), Egypt (10), Europe (1), Finland (1), France (3), Germany (9), Hong Kong (1), Hungary (1), India (14), Indonesia (1), Iran (3), Iraq (1), Israel (3), Italy (18), Japan (12), Korea (3), Latvia (1), Malaysia (4), the Netherlands (2), New Zealand (2), Oman (1), Poland (3), Portugal (4), Romania (1), Slovenia (2), South Korea (4), Spain (6), Sweden (1), Taiwan (1), Thailand (1), The Netherlands (1), Turkey (2), the United Kingdom (4), and the United States of America (55) (Table S2).

Nineteen original articles were selected for full-text review; of them, only fourteen met the inclusion criteria and were included in the present systematic review (Figure 1). We observed an agreement percentage of 96% (Fleiss’ kappa = 0.74, *p* < 0.001). 

### 3.2. Study Characteristics 

Fourteen articles studied the associations between oxidative stress genes related to bone health outcomes, such as BMD, osteopenia, osteoporosis, and osteoporotic fracture. All of them fulfilled the selection criteria, and the characteristics of these studies are illustrated in detail in Table 1. The identified papers were published from 2003 to date. Five studies were conducted in the Slovenia population [18,19,20,21,22], two studies in China [23,24], and one in India, Iran, Spain, Japan, Korea, Egypt, and the United Kingdom each [25,26,27,28,29,30,31]. Sample size widely varied, ranging from 142 to 426,824 individuals.

Thirty percent of the studies were conducted in pre or postmenopausal women [18,21,24,29], and the rest involved both men and women adults [19,20,22,23,25,26,27,28,30,31]. A total of 72% percent of all the studies were of a cross-sectional design, 21% were case-control studies, and 7% were cohort studies, mostly diagnosed according to the following criteria: osteoporosis T-score ≥ 2.5 SD below peak bone mass by DXA, except one study performed in Spain, which used peripheral instantaneous X-ray imaging [27]. Seventy-nine percent of the reports were adjusted by confounding variables, mainly age, sex, and BMI, while the rest of the papers did not report it.

Finally, we obtained 21 oxidative stress genes reported in the selected literature: Arachidonate 12-Lipoxygenase 12S type (*ALOX12*); BUD13 homolog (*BUD13*); Catalase (*CAT*); Glutathione-S reductase (*GSR*); Thioredoxin Reductase 1 (*TXNRD1*); Superoxide dismutase 1 (*SOD1*); Superoxide dismutase 2 (*SOD2*); Mannose-6-Phosphate Receptor (*M6PR*); Glutathione Peroxidase 6 (*GPX6*); Thioredoxin 2 (*TXN2*); Paraoxonase-1 (*PON1*), Paraoxonase-2 (*PON2*); Glutathione S-Transferase Mu 3 (*GSTM3*); Cytochrome P450 Family 4 Subfamily F Member 2 (*CYP4F2*); Scavenger Receptor Class B Member 1 (*SCARB1*); Nitric Oxide Synthase 3 (*eNOS*); variable-number tandem repeat (*VNTR*); Glutathione S-Transferase Mu 1 (*GSTM1*); Glutathione S-Transferase Theta 1 (*GSTT1*); Glutathione S-Transferase Pi 1 (*GSTP1*); and Glutathione Peroxidase 1 (*GPx1*). We cataloged the polymorphisms that affect bone mass (Table 2) from those that influence osteoporotic fracture (OF) (Table 3).

### 3.3. Risk of Bias

The risk of bias was estimated to be low across the whole set of studies (Tables S3–S5).

### 3.4. Results of Individual Studies and Syntheses

The association between genetic variants belonging to oxidative-stress-related genes in osteoporosis was reported in 14 articles. All the details of the genetic associations are described in Table 2 and Table 3.

#### 3.4.1. Antioxidant Enzymes

*SOD* genes family. A total of three studies have evaluated the effect of polymorphisms in the *SOD* gene related to BMD levels [18,23,25]. Mlakar, in 2012, analyzed the variants rs4998557 (SOD1int1(G > A)) in *SOD1* and rs4880 (SOD2_Ala16Val) in *SOD2* with the BMD levels in a postmenopausal women cohort from Slovenia without a significant association founded [18]. However, previously, Deng and colleagues evaluated several SNVS in a Chinese population as follows: rs11968525-C; rs2053949-T; rs7754103-G; rs7754295-G; rs12192410-A; rs10455776-C; rs12525670-C; and rs9355741-G in the *SOD2* gene. Three SNVs (rs7754103, rs7754295, and rs2053949) were significantly associated with the expression level of the *SOD2* gene represented by at least one probe (*p* < 0.05). The strongest association with high BMD was observed in rs11968525-C SNV (β = 9.50 × 10^−3^, *p* = 0.048) [23]. Additionally, a study performed by Botre and colleagues in an Asian Indian population described an increased odds ratio between the allele rs4880-C with osteoporosis (OR = 1.50, *p* < 0.05; χ2 = 19.908, *p* < 0.0001). Contrary to rs5746094-G in *SOD2*, that was related to a protective association (OR = 0.23, *p* < 0.05; χ2 = 24.6206, *p* < 0.001) with osteoporosis [25].

*PON* genes family. Yamada and colleagues in 2003 showed that rs662 (Gln192Arg) and rs854560 (Leu55Met) SNVs in *PON1* and rs7493 (Cys311Ser) in *PON2* are associated with low BMD values for lumbar spine (*p* < 0.05) and femoral neck (*p* < 0.05) in a population of Japanese postmenopausal women. The authors suggested that those variants are risk factors for reduced bone mass [28].

*GPx* genes family. Two studies have analyzed SNVs in the *GPx* gene family related to BMD values. Mlakar, in 2010, examined rs1050450 (Pro198Leu) and PolyAla polymorphism on the *GPx1* gene in a Slovenia population. Their results showed that individuals carrying the C/C -rs1050450 genotype were significantly associated with higher BMD values of the femoral neck and total hip (*p* < 0.026 and *p* < 0.023, respectively). Additionally, subjects carrying a minor homozygous genotype T/T of PolyAla were associated with lower BMD values in the lumbar spine and total hip (*p* = 0.032 and *p* = 0.018, respectively) [22]. Recently, Usategui-Martin and colleagues, reported that rs406113-C and rs974334-G variants on the *GPx6* gene are genetic risk factors for osteoporotic fracture (OR = 1.68, *p* < 0.001 and OR = 1.69, *p* = 0.002, respectively) in a Caucasic population [27]. 

*GST* gene family. Polymorphism in the *GST* gene family has been reported in three independent studies [19,20,21]. Mlakar, in 2011, reported a borderline significant association between rs74837985-C (Lys173Asp) in the *GSTM1* gene with BMD lumbar spine values (*p* = 0.100). In contrast, the rs11550605-A (Thr104Pro) variant in *GSTT1* was associated with higher BMD measures in the femoral neck (*p* = 0.023), lumbar spine (*p* = 0.017), and total hip (*p* = 0.031) in a Slovenian elderly population [20]. Later, the same authors examined the association between the functional *GSTM3* gene polymorphism, rs7483 (Val224Ile), and rs1799735 (224Ile-insAGG) with BMD values in a Slovenian population. Carriers with at least one Ile allele compared to Val/Val homozygotes of *GSTM3* were significantly related to the variation in BMD total hip (0.831 ± 0.148/0.859 ± 0.125 vs. 0.858 ± 0.139, *p* = 0.012). No significant differences in BMD values between genotype subgroups of insAGG polymorphisms were found [19]. Later, in 2012, Mlakar et al. analyzed rs1138272 (Ala114Val) and rs1695 (Ile105Val) polymorphism in the *GSTP1* gene in 523 Slovenian pre- and post-menopausal women. These results showed significant border differences between the 114Val and 105 Val carriers associated with BMD values in osteopenic postmenopausal women [21].

*TXN* gene family. Usategui-Martin and colleagues found that the rs4964779-C allele and rs4077561-T in the *TXNRD1* gene are two genetic risk factors associated with osteoporotic fracture (OR = 1.92, *p* < 0.001 and OR = 1.48, *p* = 0.002, respectively). In addition, the rs2281082-T allele in the *TXN2* gene was associated with a reduced risk of osteoporotic fracture in the HORTEGA study cohort [27].

*CAT* gene. The *CAT* gene has been analyzed in two independent studies [18,29]. Oh et al. evaluated the association between several polymorphisms in the CAT gene and BMD levels in a Korean postmenopausal women cohort. After adjusting for confounders, a significant association was found between rs17880449 (+22348C→T) and higher BMD at the lumbar spine (*p* = 0.010) and femoral neck (*p* = 0.050). Otherwise, the polymorphisms rs17881315 (−20T→C), rs17886119 (+144C→T), and rs17879188 (+33078A→G) did not reach significant association [29]. Additionally, Mlakar in 2012 analyzed the rs511895 (CATint10T>C) variant in the *CAT* gene in a postmenopausal women cohort from Slovenia without a significant association related to BMD values [18]. 

*ALOX12*. Al-e-Ahmad and colleagues showed that homozygous carriers for the rs2292350-A allele had lower BMD mean femoral neck (0.72 ± 0.13) than the G-homozygous allele (0.90 ± 0.18) in an Iranian elderly population study. However, the rs9897850 variant was not significative associated with BMD values [26].

*GSR.* Mlakar and colleagues analyzed the rs2978663 (GSRint3(A>G)) and rs2911678 (GSRint10(T>A) variants in the *GSR* gene and their association with the BMD values in a Slovenian postmenopausal women cohort [18]. Significant associations were detected between the rs2978663-G allele and BMD variation at all measured skeletal sites in women with at least one G allele compared with AA homozygous (femoral neck, *p* = 0.044; lumbar spine, *p* = 0.043; and total hip skeletal, *p* = 0.009). In contrast, the rs2911678 polymorphism did not reach a significant association [18].

*NOS* gene. Liu and colleagues investigated the relationship between *eNOS* G894T and 27 bp variable-number tandem repeat (*VNTR*) gene polymorphism and osteoporosis in a Chinese menopausal women population [24]. Their results showed that the average BMD values of the femoral neck, ward’s triangle, and lumbar vertebrae 1~4 (L1~L4) in subjects with T/T genotype in eNOS G894T were significantly higher than those in the subjects with G/T and G/G genotypes (*p* < 0.05). In addition, the average BMD of the femoral neck in the subjects with a/a genotype of eNOS 27 bp-VNTR was higher than that in non-carriers (*p* < 0.004) [24]. 

#### 3.4.2. Vitamin-Metabolism-Related Genes

α-tocopherol-related genes. One previous study explored three SNVs related to α-tocopherol levels and the variation of BMD in a population from Sweden and the UK. These SNVs included rs964184 close to *BUD13*, *ZNF259*, and *APOA1/C3/A4/A5*; rs2108622 close to *CYP4F2*; and rs11057830 close to *SCARB1*. Two of the three variants (rs2108622-T and rs11057830-A alleles) were strongly associated with higher BMD values and fracture risk (OR = 0.11, *p* < 0.001, and OR = 0.10, *p* < 0.001, respectively). However, no significant differences in BMD according to the rs964184-G were found [31].

#### 3.4.3. Others

*M6PR*. Usategui and colleagues analyzed whether the rs1805754 polymorphism on the *M6PR* gene could modify the osteoporotic fracture risk in the HORTEGA follow-up study. They found that the rs1805754-C allele was an important genetic risk factor for fracture (OR = 2.14, *p* < 0.001) [27].

## 4. Discussion 

This systematic review investigated the potential effect of oxidative-stress-related genes on the pathophysiology of osteoporosis by qualitative synthesis of information derived from 14 original research papers. The analyzed polymorphisms belonging to a wide variety of genes, e.g., *SOD*, *PON*, *GPx*, *GST*, *TXN*, *CAT*, *ALOX12*, *GSR*, and *NOS*, focused on BMD variation, which is the current clinical gold standard for analyzing reductions in bone mass and diagnosing osteoporosis.

SOD is one of the major antioxidant enzymes encoded by a broad *SOD* gene family [32]. SOD plays a vital role in the clearance of ROS by its capacity to enzymatically dismutate superoxide into hydrogen peroxide, contributing to the control of oxidative stress in normal cells [33]. Evidence suggests that ROS are involved in bone resorption, directly contributing osteoclast-generated superoxide to bone degradation [34]. While the exact mechanism by which ROS accelerates bone resorption is still unclear, ineffective neutralization of ROS leading to oxidative stress in the bone can increase bone loss and bone weakness, typical of osteoporosis [35]. According to published studies, *SOD* variants are significantly associated with BMD levels in Indian and Chinese populations [18,23,25]. The rs4880 and rs5746094 SNVs represent a risk factor for low BMD values, contrary to rs11968525, related to higher BMD in middle-aged Chinese people [23].

The human *PON1* gene, located on the long arm of chromosome 7 (q21.22), encodes the PON1 enzyme. PON1 is a calcium-dependent esterase closely associated with high-density lipoproteins (HDL) and confers antioxidant properties by preventing the accumulation of lipid peroxidation products [36]. Lipid peroxidation products can inhibit the differentiation of osteoblasts by changing mineral content, decreasing bone formation, and inhibiting mineralization; therefore, this condition may cause osteoporosis [35]. Previous studies revelated that some polymorphisms in this gene may influence PON activity [37]. The findings about variants in the *PON* gene suggest a key role in the BMD variation determined in Japanese postmenopausal women. Therefore, PON1 SNVs may be critical in bone metabolism and osteoporosis [38].

The *GPX* gene family encodes the antioxidant enzyme GPX, which catalyzes the degradation of peroxides and hydroperoxides by oxidizing glutathione, significantly regulating oxidative stress [39]. Osteoclast-generated superoxides participate in bone degradation and stimulate osteoclast differentiation and RANKL expression. Therefore, it is involved in bone degradation [40]. As coupled mechanisms, osteoblasts produce enzymatic antioxidants such as GPX1, preventing cellular injury [41]. Additionally, decreased GPX1 enzyme activity and the development of osteoporosis have been established in postmenopausal women [35]. The polymorphisms reported in the literature have been significantly associated with the variability in the BMD of the femoral neck and total hip in a Slovenian population, representing a genetic risk factor for osteoporotic fracture in a Caucasian population.

*GST*s are a supergene family of dimeric enzymes that catalyze the conjugation of glutathione [42]. This intracellular solute protects against oxidative damage of endogenous and exogenous reactive metals and electrophiles [43]. Mainly, GST1 interacts with Jun N-terminal kinase (JNK), a member of stress-activated Ser/Thr kinases, which is involved in late-stage osteoblast differentiation [44]. Likewise, the activation of the FGF-2/MEK/ERK1/2/Akt/p70(S6K)/NF-B and PKC/JNK pathways may contribute to the recruitment of osteoblasts [44]. Although Mlakar and colleagues (2012) have demonstrated that GST genes are associated with BMD, their role in the association between polymorphisms in the GSR gene and osteoporosis susceptibility remains unclear. 

The thioredoxin (Txn) pathway is a cellular antioxidant system that regulates the cells’ redox status [45]. Txn 1 (Txn1) and 2 (Txn2) are redox protein essential for controlling ROS homeostasis, apoptosis, and cell viability [46]. It has been reported that genetic alterations in *TXN2* are associated with impaired mitochondrial function and increased oxidative stress [47]. *TXN* is expressed in osteoblasts, osteoclasts, and chondrocytes and affects the differentiation and functioning of skeletal cells through redox-dependent mechanisms. *TXN* expression is reduced during human osteoclast differentiation induced by a soluble nuclear factor-kβ ligand-receptor activator (sRANKL) and macrophage colony-stimulating factor (M-CSF) [48]. The findings report that the genetic variants in genes involved in the Txn pathway confirm that they could have a crucial role in osteoporotic fracture.

The *CAT* gene is located on chromosome 11 (11p13) and encodes the CAT enzyme [29]. The CAT enzyme converts hydrogen peroxide into molecular oxygen and water, thus preventing the accumulation of hydrogen peroxide that fuels aging, inflammation, and cancer [49]. *CAT* is strongly upregulated during osteogenic differentiation of mesenchymal stem/stromal cells (MSCs) [50]. Regulated CAT activity induces osteogenic differentiation of vascular smooth muscle cells by increasing Runt-related transcription factor 2 (Runx2), a key transcription factor for osteogenesis [51]. Among the polymorphisms analyzed, only rs17880449 was associated with higher BMD at the lumbar spine in the Slovenian cohort studied [29]. Hence, *CAT* polymorphisms linked to osteoporosis remain controversial, making it necessary to increase the study sample and explore its functional implication with BMD variation. Nevertheless, a Korean search group showed a strong association of *CAT* SNVs with osteonecrosis of the femoral head from various causes, which sheds light on the role of *CAT* polymorphisms in bone metabolism [52].

ALOX12 enzyme, encoded by the *ALOX12* gene, produces lipid peroxides, leading to oxidative stress and the development of osteoporosis [53,54]. ALOX12 belongs to the arachidonate lipoxygenase enzyme superfamily, which catalyzes the insertion of molecular oxygen into polyunsaturated fatty acids, such as arachidonic acid [55]. The product of ALOX12 activity, i.e., 12-hydroperoxyeico-satetraenoic acid (12-HPETE), acts as an endogenous ligand for the peroxisome proliferator-activated receptors (PPARs), which inhibit osteoblastogenesis and increase adipogenesis from a common progenitor, namely mesenchymal stem cells (MSCs) [56]. Therefore, ALOX12 activation could result in the upregulation of the pathway of PPARs, decreasing osteoblastogenesis and BMD [57]. The SNVs in *ALOX12* reportedly influenced the BMD variations in the Iranian cohort study, contributing to the development of osteoporosis.

GSR belongs to the glutathione recycling system, which reduces oxidized glutathione [58]. Previous studies have indicated that decreased antioxidant enzyme activity, such as GSR, might cause markedly increased bone demineralization [59]. GSR was found to be upregulated in an osteosarcoma cell line, as well as a decreased cell proliferation rate in osteoblasts from osteoporotic tissue [41]. In contrast, GSR activity remains unaltered in postmenopausal osteoporosis women [40]. Moreover, Mlakar and colleagues demonstrated that rs2978663 (GSRint3(A>G)) polymorphism is associated with BMD variation at several sites measured, indicating that this polymorphism could influence BMD in the Slovenian women population [18]. 

The human *eNOS* gene at 7q35-36 includes 26 exons and 25 introns, encoded to NOS isozymes. Three NOS isozymes, neuronal NOS (nNOS), inducible NOS (iNOS), and endothelial NOS (eNOS), have been described in mammalians, even though the eNOS isoform is predominant in bone cells [60]. The NOS enzyme synthesizes nitric oxide (NO), a signaling molecule produced from L-arginine [61]. It has been found that NO may modify skeletal remodeling by reducing the formation and differentiation of osteoblast and osteoclast [62]. Intracellular NO in pre-differentiated and differentiated osteoblasts stimulate aerobic glycolysis, considering the major energy resource in differentiated osteoblasts [63]. Exogenous NO liberated by osteoblasts and osteocytes avoids osteoclast attachment and resorption. Conversely, NO produced by osteoclasts has dual effects on osteoclasts, depending on constitutive or inducible NOS activity [64]. IFN-γ induction of NOS by cells in the marrow leads to the suppression of osteoclastogenesis, whereas constitutive NOS activity stimulates bone resorption [65]. Furthermore, granulocyte colony-stimulating factor induces neutrophils to produce NO, inhibiting osteoblast differentiation [66]. Additionally, it has been described that NO was upregulated in osteoblasts by estrogen in a cell-autonomous manner and could drive osteoblast proliferation and differentiation [67]. Therefore, NO has been associated with osteoporosis in postmenopausal women [35]. eNOS also could mediate the effects of mechanical loading on the skeleton, where it acts along with prostaglandins to regulate bone formation [61]. It has been described that *eNOS* polymorphisms can change the activity of the eNOS enzyme, thereby changing NO concentration in tissues and impacting BMD variation [24].

α-Tocopherol has the highest antioxidant biological activity and is the most abundant form of vitamin E [68]. Current evidence of vitamin E supplementation reveals beneficial properties against osteoporosis through various pathways [69]. For example, it prevents bone calcium loss by scavenging free radicals. Vitamin E plays an essential role in oxidative stress signaling with effects on the receptor activator of nuclear factor kappa-B (RANK)/receptor activator of nuclear factor kappa-B ligand (RANKL)/osteoprotegerin (OPG), and Wnt/β-catenin systems, affecting osteoclast and osteoblast activity [70]. Furthermore, vitamin E has been reported to be anti-inflammatory in preventing osteoporosis, regulating cytokines, such as IL-1, IL-6, RANKL, OPG, and M-CSF, critical determinants of osteoclast differentiation, and bone resorptive activity [71]. 

*BUD13*, *CYP4F2*, and *SCARB1* genes have been associated with lipid traits in European populations [72,73,74]. However, a previous GWAS found strong evidence between variants near *BUD13* and *ZNF259*, and *APOA1/C3/A4/A5*, *CYP4F2*, and *SCARB1* cluster genes are associated with circulating α-tocopherol levels [75]. To analyze the association between α-tocopherol gene-related BMD variation, Michaelson and colleagues measured SNVs close to this gene cluster in a population from Sweden and the UK, with a significative association with high BMD [31]. CYP4F2 is a cytochrome P450 monooxygenase family member, identified as the enzyme that hydroxylates c-tocopherol and leukotrienes [76]. On the other hand, the scavenger receptor class B type 1 (*SCARB1*) gene is a glycosylated cell-surface receptor for HDL. It thus plays a vital role in lipid metabolism. In osteoblastic cells, SR-B1 is implicated in the selective uptake of cholesterol and estradiol from LDL and HDL [77]. Thus, SR-B1 represents a possible correlation between atherosclerosis and osteoporosis, suggesting that *SCARB1* contributes to the regulation of bone metabolism [78].

Bone resorption mediated by osteoclasts depends partly on lysosome enzymatic activity [79]. Many of the acid hydrolases required are transported from the Golgi apparatus to lysosomes after acquiring a N-glycan linked mannose 6 phosphate (M6P) recognition mark [80]. The cation-dependent M6P receptor, encoded by the *M6PR* gene (Chr 12p13.31), recognizes these M6P residues, mediating the transference of hydrolases to the lysosomal compartments; however, its direct participation in bone metabolism has not been established yet [81]. Nevertheless, it has been postulated that a deficiency of the M6P tag missorts the distribution of the lysosomal enzymes, increasing bone resorption [82,83]. Usategui-Martín and colleagues reported that the rs1805754-C variant located in the promoter region of *M6PR* increases the risk of osteoporotic fracture; therefore, variation in *M6PR* gene expression could result in lysosomal dysfunction and impaired bone remodeling [27].

This systematic review highlights promising candidate SNVs in oxidative stress pathway-related genes that might impact BMD variation and lead to osteoporosis. However, it should be noted that heterogeneity in the selected studies represents a potential limitation, which also caused the decision to refrain from conducting a meta-analysis. Despite this limitation, the strength lies in the detailed information on individual SNVs and their association with bone health traits. Furthermore, this is the first systematic review to summarize oxidative-stress-related polymorphisms, highlighting their role in this complex disease. Further research, preferably more extensive cohort studies, are needed to delineate the accuracy of genetic variants in the development of osteoporosis. In addition, we strongly recommend designing studies that include adjustment for confounding variables, such as gender and age, which have been reported to influence the interpretation of results. Therefore, delving into their specific genetic mechanisms represents a promising research area that opens the doors to future lines of research to apply this knowledge in clinical practice.

## 5. Conclusions

Oxidative stress is involved in the development of osteoporosis and its associated comorbidities. The utility of assessment polymorphisms in genes regulating redox balance remains to be explored in future prospective cohort studies. However, the role that oxidative stress plays in the pathophysiology of osteoporosis is indisputable, and understanding the mechanisms by which it contributes to the initiation and maintenance of this disease can aid the management in the near future. Thus, SNVs may emerge as essential tools in the early diagnosis of osteoporosis, from the subclinical stage, in evaluating the pattern of evolution and the therapeutic response. 

## Figures and Tables

**Table 1:** Summary of studies included in the systematic review.

Country	Study Design	Sample Size (W/M)	Number of Cases	Number of Controls	Mean Age (Years)	Measurement Site (BMD)	Outcome	Confounders	Author, Year
India	Cross-sectional study	180	98	82	Case: 60.20 ± 7.42 Control: 57.4 ± 8.51	Peripheral-calcaneus BMD (spine or proximal femur)	OS ^a^	NR	Botre et al., 2015 [25]
Iran	Case-control study	180 (90 W/90 M)	90 (45 W/45 M)	90 (45 W/45 M)	Case: (W: 66.04 ± 4.88; M: 69.73 ± 6.83) Control: (W: 64.93 ± 4.69; M: 68.11 ± 6.36)	LS and proximal FN	OS ^b^	Age, gender	Al-E-Ahmad et al., 2018 [26]
China	Cross-sectional study	1627 (825 W/802 M)	1627	-	34.5 ± 13.2	Total hip (FN, trochanter, and intertrochanter region)	BMD	Age, gender, height, and weight	Deng et al., 2011 [23]
Slovenia	Cross-sectional study	468	468	-	63.71 ± 8.279	LS, TH, FN	BMD	Age, BMI, year since menopause	Mlakar et al., 2012 [18]
Spain	Case-control study	575 (296 W/279 M)	221 (189 W/165 M)	354 (107 W/114 M)	Cases: 61.37 ± 17.88; Control: 61.88 ± 16.32	Right calcaneus	OF ^c^	Sex, age, BMI, BMD, menopause, hypertension and family history of OF.	Usategui-Martín et al., 2022 [27]
Japan	Cross-sectional study	2119 * (1087–1094 W/1112–1125 M)	-	-	40–79	LS and right FN	BMD	Age	Yamada et al., 2003 [28]
Korea	Cross-sectional study	560	560	-	59.4 ± 7.2	LS, FO	BMD, OF	Age, year since menopause, weight, and height	Oh et al., 2007 [29]
Slovenia	Cross-sectional study	712 (593 W/119 M)	712	-	W:60.84 ± 9.99; M:67.92 ± 5.93	LS, TH, FO	BMD ^d^	Age, height, and weight	Mlakar et al., 2012 [19]
Egypt	Case-control study	142 (124 W/18 M)	97 (85W/12M)	45 (39W/6M)	Case: 47.9 ± 8.9; Control: 45.9 ± 8.1	LS, FO	OS, OF ^e^	NR	Senosi et al., 2022 [30]
Sweden (UK biobank)	Cross-sectional analysis	426,824	53,184	373,611	NR	Heel	BMD, OF	BMI, fat-free soft tissue body mass, height, type 2 diabetes, smoking, alcohol consumption, and steroid hormones.	Michaëlsson et al., 2021 [31]
China	Case-control study	281	123 OP/127 OS	31	45 to 65	LS, left hips	BMD ^b^	NR	Liu et al., 2009 [24]
Slovenia	Cross-sectional study	712 (593 W/119 M)	292 (266 W/26 M)	420 (327 W/93 M)	62.04 ± 9.79	LS, TH, FO	BMD ^d^	Sex, BMI, age	Mlakar et al., 2011 [20]
Slovenia	Cohort	523	468	55	63.71 ± 8.28; 44.56 ± 3.9	LS, TH, FO	BMD ^d^	Age, height, weight, years since menopause, smoking status and glucocorticoid use	Mlakar et al., 2012 [21]
Slovenia	Cross-sectional study	682 (571 W/111 M)	682	-	62.4 ± 9.79	LS, TH, FO	BMD ^b^	Age, weight, height and BMI	Mlakar et al., 2010 [22]

Abbreviations: BMI: bone mineral density; OP: osteoporosis; OF: osteoporotic fracture; OS: osteopenia; N: normal; W: women; M: men; NR: not reported; BMI: body mass index; LS = lumbar (L1-L4) spine; TH: total hip; FN: femoral neck; FO = forearm; ^a^: BMD, normal as T-score ≥ 1.1 SD; osteoporotic as T-score ≤ 2.5 SD; ^b^: BMD WHO criteria (cases: T-score ≤ 2.5 SD; controls: T-score ≥ 1.0 SD); ^c^: Genant classification; ^d^: International Society for Clinical Densitometry (ISCD) criteria; ^e^: American College of Rheumatology/European League Against Rheumatism criteria. Osteoporotic fractures were determined after a follow-up of 12–14 years; * varied in each SNV analyzed.

**Table 2:** Polymorphisms related to oxidative stress, BMD, and osteoporosis risk.

Gene	Chr	Significant SNV	Main Findings	Non-Significant SNV	Outcome	Reference
Antioxidant enzymes						
*SOD1*	*21*			rs4998557	↑BMD	Mlakar et al., 2012. [18]
*SOD2*	6			rs4880	↑BMD	Mlakar et al., 2012. [18]
		rs11968525	9.50 × 10^−3^, *p* = 0.048 ^A^	rs2053949, rs7754103, rs7754295, rs12192410, rs10455776, rs12525670 rs9355741	↑BMD	Deng et al., 2011. [23]
		rs4880	1.50 (0.85–2.63) ^B,^ *p* < 0.05		↑OP	Botre et al., 2015 [25]
		rs5746094	0.23 (0.11–0.30) ^B^ **, *p* < 0.05		↑OP	
*PON1*	7	rs662	Femoral neck 0.633 ± 0.005 vs. 0.653 ± 0.004, *p* < 0.001 ^C^ Lumbar spine 0.798 ± 0.007 vs. 1.034 ± 0.012, *p* <0.05 ^PMW^		↓BMD ^PMW^	Yamada et al., 2003. [28]
		rs854560	Femoral neck 0.640 ± 0.003 vs. 0.774 ± 0.006, *p* < 0.01 ^a^ Lumbar spine 0.803 ± 0.005 vs. 0.848 ± 0.013, *p* < 0.01 ^a^			
*PON2*		rs7493	Femoral neck 0.638 ± 0.004 vs. 0.657 ± 0.005, *p* < 0.05 ^a^		↓BMD ^PMW^	
*GPX1*	3	rs1050450	Femoral neck, *p* < 0.026 ^NA^ Total hip, *p* < 0.023 ^NA^		↓BMD	Mlakar et al., 2010. [22]
		PolyAla region	Lumbar spine, *p* = 0.032 ^NA^ Total hip, *p* = 0.018 ^NA^		↓BMD	
*GSTM1*	1			*GSTM1^-/-^* rs74837985	↑ BMD	Mlakar et al., 2011. [20]
*GSTT1*		*GSTT1^-/-^* rs11550605	Femoral neck, *p* = 0.023 ^NA^ Lumbar spine *p* = 0.017 ^NA^ Total hip, *p* = 0.031 ^NA^		↑ BMD	Mlakar et al., 2011. [20]
*GSTM3*	1	rs7483	Total hip ^VDB^ 0.831 ± 0.148/0.859 ± 0.125 vs. 0.858 ± 0.139 *p* = 0.012 ^C^	rs1799735	↑BMD	Mlakar et al., 2012. [19]
*GSTP1*	*11*			rs1138272 rs1695	BMD	Mlakar et al., 2012. [21]
*CAT*	*11*	rs17880449	Lumbar spine ^VDB^ 0.850 ± 0.170/0.880 ± 0.180 vs. 0.880 ± 0.150 *p* = 0.010 ^C^ Femoral neck ^VDB^ 0.710 ± 0.130/0.730 ± 0.120 vs. 0.730 ± 0.120 *p* = 0.050 ^C^	rs17881315, rs17879188, rs17886119	↑BMD	Oh et al., 2007 [29]
				rs511895	↑BMD	Mlakar et al., 2012. [18]
*ALOX12*	*17*	rs2292350	9.15 (1.06–79.11) ^B^, *p* = 0.044 ^NOW^	rs9897850	↑OP	Al-E-Ahmad et al., 2018. [26]
*GSR*	*8*	rs2978663	Femoral neck ^VDB^ 0.689 ± 0.12/0.702 ± 0.132 vs. 0.712 ± 0.110 *p* = 0.044 ^C^ Total hip ^VDB^ 0.826 ± 0.144/0.849 ± 0.152 vs. 0.863 ± 0.107 *p* = 0.009 ^C^ Lumbar spine ^VDB^ 0.847 ± 0.167/0.870 ± 0.168 vs. 0.880 ± 0.163 *p* = 0.043 ^C^	rs2911678	↑BMD	Mlakar et al., 2012. [18]
*eNOS*	7	rs1799983	Femoral neck ^VDB^ 0.714 ± 0.109/0.717 ± 0.099 *vs*. 0.817 ± 0.143, *p* < 0.05 ^C^		↓OP	Liu et al., 2009 [24]
		27bp-VNTR	0.29 (0.11-0.77) ^B^, *p* < 0.004		↓OP	Liu et al., 2009 [24]

^A^: β Values of association; ^B^: Odds ratio estimation; ^C^: *p*-value obtained using a dominant statistic model (less common homozygotes together with heterozygotes were compared with common homozygotes). Increase (↑) and Decrease (↓) the outcome risk. Comparison with premenopausal women (PMW) or non-osteoporotic women (NOW), NA: not reported. *P*-values were obtained through comparison mayor allele homozygote vs. (^a^) heterozygote in postmenopausal women. Values are means ± SE of bone mineral density (BMD). VDB: Values BMD using dominant model (the less common homozygotes/heterozygotes) vs. common homozygotes. ** Data were re-calculated because a mistake was detected in the original paper.

**Table 3:** Polymorphisms related to oxidative stress and osteoporotic fracture risk.

Gene	Chr	Significant SNV	Main Findings	Non-Significant SNV	Outcome	Reference
Antioxidant enzymes						
*GPX6*	6	rs406113	1.68 (1.30–2.16) ^A^, *p* < 0.001 ^FR^		↑OF	Usategui-Martin et al., 2021. [27]
		rs974334	1.69 (1.21–2.35) ^A^, *p* = 0.002 ^FR^		↑OF	Usategui-Martin et al., 2021. [27]
*TXNRD1*	*12*	rs4964779	1.92 (1.38–2.66) ^A^, *p* < 0.001 ^B^		↑OF	Usategui-Martin et al., 2021. [27]
		rs4077561	1.48 (1.16–1.89) ^A^, *p* = 0.002 ^B^		↑OF	Usategui-Martin et al., 2021. [27]
*TXN2*	*22*	rs2281082	0.49 (0.41–0.67) ^A^, *p* < 0.001 ^B^		↓OF	Usategui-Martin et al., 2021. [27]
Vitamin-metabolism-related genes						
*BUD13/ZNF259/* *APOA5*	11			rs964184	↑BMD; OF	Michaëlsson et al., 2021. [31]
*CYP4F2*	19	rs2108622	0.11 (0.07–0.14) ^A^, *p* < 0.001 ^B^		↑BMD; OF	Michaëlsson et al., 2021. [31]
*SCARB1*	12	rs11057830	0.10(0.06–0.15) ^A^, *p* < 0.001 ^B^		↑BMD; OF	Michaëlsson et al., 2021. [31]
Others						
*M6PR*	*12*	rs1805754	2.14 (1.61–2.86) ^A^, *p* < 0.001 ^B^		↑OF	Usategui-Martin et al., 2021. [27]

^A^: Odds ratio estimation; ^B^: *p*-value obtained using a dominant statistic model (less common homozygotes together with heterozygotes were compared with common homozygotes). Increase (↑) and Decrease (↓) the outcome risk. Comparison with osteoporotic fracture (OF) group. Values are means ± SE of bone mineral density (BMD). FR: Fracture.

## Data Availability

The datasets analyzed in this study are available in the supplementary material and could be available from the corresponding author.

## Supplementary Materials

The following supporting information can be downloaded at: https://www.mdpi.com/article/10.3390/antiox12040915/s1, Table S1: Search strategy in the website database; Table S2: Excluded articles; Table S3: The Joanna Briggs Institute Critical Appraisal Checklist for analytical cross-sectional studies; Table S4: The Joanna Briggs Institute Critical Appraisal Checklist for case-control studies; Table S5: The Joanna Briggs Institute Critical Appraisal Checklist for cohort studies.

## References

[1] Yong E.L., Logan S. (2021). Menopausal Osteoporosis: Screening, Prevention and Treatment. Singap. Med. J..

[2] Fischer V., Haffner-Luntzer M. (2022). Interaction between Bone and Immune Cells: Implications for Postmenopausal Osteoporosis. Semin. Cell Dev. Biol..

[3] Albergaria B.H., Chalem M., Clark P., Messina O.D., Pereira R.M.R., Vidal L.F. (2018). Consensus Statement: Osteoporosis Prevention and Treatment in Latin America-Current Structure and Future Directions. Arch. Osteoporos..

[4] Prentice A. (2001). The Relative Contribution of Diet and Genotype to Bone Development. Proc. Nutr. Soc..

[5] Raggatt L.J., Partridge N.C. (2010). Cellular and Molecular Mechanisms of Bone Remodeling. J. Biol. Chem..

[6] Pisoschi A.M., Pop A. (2015). The Role of Antioxidants in the Chemistry of Oxidative Stress: A Review. Eur. J. Med. Chem..

[7] Bhatti J.S., Bhatti G.K., Reddy P.H. (2017). Mitochondrial Dysfunction and Oxidative Stress in Metabolic Disorders-A Step towards Mitochondria Based Therapeutic Strategies. Biochim. Biophys. Acta Mol. Basis Dis..

[8] Valko M., Leibfritz D., Moncol J., Cronin M.T.D., Mazur M., Telser J. (2007). Free Radicals and Antioxidants in Normal Physiological Functions and Human Disease. Int. J. Biochem. Cell Biol..

[9] Alkadi H. (2020). A Review on Free Radicals and Antioxidants. Infect. Disord. Drug Targets.

[10] Kaur G., Sharma A., Bhatnagar A. (2021). Role of Oxidative Stress in Pathophysiology of Rheumatoid Arthritis: Insights into NRF2-KEAP1 Signalling. Autoimmunity.

[11] Banfi G., Iorio E.L., Corsi M.M. (2008). Oxidative Stress, Free Radicals and Bone Remodeling. Clin. Chem. Lab. Med..

[12] Yuan Y., Yang J., Zhuge A., Li L., Ni S. (2022). Gut Microbiota Modulates Osteoclast Glutathione Synthesis and Mitochondrial Biogenesis in Mice Subjected to Ovariectomy. Cell Prolif..

[13] López-Armada M.J., Fernández-Rodríguez J.A., Blanco F.J. (2022). Mitochondrial Dysfunction and Oxidative Stress in Rheumatoid Arthritis. Antioxidants.

[14] Trajanoska K., Rivadeneira F. (2019). The Genetic Architecture of Osteoporosis and Fracture Risk. Bone.

[15] Rivadeneira F., Mäkitie O. (2016). Osteoporosis and Bone Mass Disorders: From Gene Pathways to Treatments. Trends Endocrinol. Metab..

[16] Shamseer L., Moher D., Clarke M., Ghersi D., Liberati A., Petticrew M., Shekelle P., Stewart L.A., Altman D.G., Booth A. (2015). Preferred Reporting Items for Systematic Review and Meta-Analysis Protocols (PRISMA-P) 2015: Elaboration and Explanation. BMJ.

[17] Moola S., Munn Z., Sears K., Sfetcu R., Currie M., Lisy K., Tufanaru C., Qureshi R., Mattis P., Mu P. (2015). Conducting Systematic Reviews of Association (Etiology): The Joanna Briggs Institute’s Approach. Int. J. Evid. -Based Healthc..

[18] Mlakar S.J., Osredkar J., Prezelj J., Marc J. (2012). Antioxidant Enzymes GSR, SOD1, SOD2, and CAT Gene Variants and Bone Mineral Density Values in Postmenopausal Women: A Genetic Association Analysis. Menopause.

[19] Mlakar S.J., Prezelj J., Osredkar J., Marc J. (2012). BMD Values and GSTM3 Gene Polymorphisms in Combination with GSTT1/GSTM1 Genes: A Genetic Association Study in Slovenian Elderly. Gerontology.

[20] Mlakar S.J., Osredkar J., Prezelj J., Marc J. (2011). Opposite Effects of GSTM1--and GSTT1: Gene Deletion Variants on Bone Mineral Density. Dis. Markers.

[21] Mlakar S.J., Prezelj J., Marc J. (2012). Testing GSTP1 Genotypes and Haplotypes Interactions in Slovenian Post-/Pre-Menopausal Women: Novel Involvement of Glutathione S-Transferases in Bone Remodeling Process. Maturitas.

[22] Mlakar S.J., Osredkar J., Prezelj J., Marc J. (2010). The Antioxidant Enzyme GPX1 Gene Polymorphisms Are Associated with Low BMD and Increased Bone Turnover Markers. Dis. Markers.

[23] Deng F.Y., Lei S.F., Chen X.D., Tan L.J., Zhu X.Z., Deng H.W. (2011). An Integrative Study Ascertained SOD2 as a Susceptibility Gene for Osteoporosis in Chinese. J. Bone Miner. Res..

[24] Liu S.Z., Yan H., Hou W.K., Xu P., Tian J., Tian L.F., Zhu B.F., Ma J., Lu S.M. (2009). Relationships between Endothelial Nitric Oxide Synthase Gene Polymorphisms and Osteoporosis in Postmenopausal Women. J. Zhejiang Univ. Sci. B.

[25] Botre C., Shahu A., Adkar N., Shouche Y., Ghaskadbi S., Ashma R. (2015). Superoxide Dismutase 2 Polymorphisms and Osteoporosis in Asian Indians: A Genetic Association Analysis. Cell. Mol. Biol. Lett..

[26] Al-e-Ahmad A., Parsian H., Fathi M., Faghihzadeh S., Hosseini S.R., Nooreddini H.G., Mosapour A. (2018). ALOX12 Gene Polymorphisms and Serum Selenium Status in Elderly Osteoporotic Patients. Adv. Clin. Exp. Med..

[27] Usategui-Martín R., Pérez-Castrillón J.L., Mansego M.L., Lara-Hernández F., Manzano I., Briongos L., Abadía-Otero J., Martín-Vallejo J., García-García A.B., Martín-Escudero J.C. (2022). Association between Genetic Variants in Oxidative Stress-Related Genes and Osteoporotic Bone Fracture. The Hortega Follow-up Study. Gene.

[28] Yamada Y., Ando F., Niino N., Miki T., Shimokata H. (2003). Association of Polymorphisms of Paraoxonase 1 and 2 Genes, Alone or in Combination, with Bone Mineral Density in Community-Dwelling Japanese. J. Hum. Genet..

[29] Oh B., Kim S.Y., Kim D.J., Lee J.Y., Lee J.K., Kimm K., Park B.L., Shin H.D., Kim T.H., Park E.K. (2007). Associations of Catalase Gene Polymorphisms with Bone Mineral Density and Bone Turnover Markers in Postmenopausal Women. J. Med. Genet..

[30] Senosi M.R., Fathi H.M., Baki N.M.A., Zaki O., Magdy A.M., Gheita T.A. (2022). Bone Mineral Density, Vitamin D Receptor (VDR) Gene Polymorphisms, Fracture Risk Assessment (FRAX), and Trabecular Bone Score (TBS) in Rheumatoid Arthritis Patients: Connecting Pieces of the Puzzle. Clin. Rheumatol..

[31] Michaëlsson K., Larsson S.C. (2021). Circulating Alpha-Tocopherol Levels, Bone Mineral Density, and Fracture: Mendelian Randomization Study. Nutrients.

[32] Bresciani G., da Cruz I.B.M., González-Gallego J. (2015). Manganese Superoxide Dismutase and Oxidative Stress Modulation. Adv. Clin. Chem..

[33] Noor R., Mittal S., Iqbal J. (2002). Superoxide Dismutase–Applications and Relevance to Human Diseases. Med. Sci. Monit..

[34] Teitelbaum S.L. (2000). Bone Resorption by Osteoclasts. Science.

[35] Ozgocmen S., Kaya H., Fadillioglu E., Aydogan R., Yilmaz Z. (2007). Role of Antioxidant Systems, Lipid Peroxidation, and Nitric Oxide in Postmenopausal Osteoporosis. Mol. Cell. Biochem..

[36] Shunmoogam N., Naidoo P., Chilton R. (2018). Paraoxonase (PON)-1: A Brief Overview on Genetics, Structure, Polymorphisms and Clinical Relevance. Vasc. Health Risk Manag..

[37] Deakin S.P., James R.W. (2004). Genetic and Environmental Factors Modulating Serum Concentrations and Activities of the Antioxidant Enzyme Paraoxonase-1. Clin. Sci..

[38] Kim B.J., Kim S.Y., Cho Y.S., Kim B.J., Han B.G., Park E.K., Lee S.H., Kim H.Y., Kim G.S., Lee J.Y. (2011). Association of Paraoxonase 1 (PON1) Polymorphisms with Osteoporotic Fracture Risk in Postmenopausal Korean Women. Exp. Mol. Med..

[39] Lubos E., Loscalzo J., Handy D.E. (2011). Glutathione Peroxidase-1 in Health and Disease: From Molecular Mechanisms to Therapeutic Opportunities. Antioxid. Redox Signal..

[40] Sontakke A.N., Tare R.S. (2002). A Duality in the Roles of Reactive Oxygen Species with Respect to Bone Metabolism. Clin. Chim. Acta.

[41] Trošt Z., Trebše R., Preželj J., Komadina R., Logar D.B., Marc J. (2010). A Microarray Based Identification of Osteoporosis-Related Genes in Primary Culture of Human Osteoblasts. Bone.

[42] Nissar S., Syed Sameer A., Rasool R., Chowdri N.A., Rashid F. (2017). Glutathione S Transferases: Biochemistry, Polymorphism and Role in Colorectal Carcinogenesis. J. Carcinog. Mutagen.

[43] Hammond C.L., Lee T.K., Ballatori N. (2001). Novel Roles for Glutathione in Gene Expression, Cell Death, and Membrane Transport of Organic Solutes. J. Hepatol..

[44] de Luca A., Federici L., de Canio M., Stella L., Caccuri A.M. (2012). New Insights into the Mechanism of JNK1 Inhibition by Glutathione Transferase P1-1. Biochemistry.

[45] Pannala V.R., Dash R.K. (2015). Mechanistic Characterization of the Thioredoxin System in the Removal of Hydrogen Peroxide. Free Radic. Biol. Med..

[46] Holzerova E., Danhauser K., Haack T.B., Kremer L.S., Melcher M., Ingold I., Kobayashi S., Terrile C., Wolf P., Schaper J. (2016). Human Thioredoxin 2 Deficiency Impairs Mitochondrial Redox Homeostasis and Causes Early-Onset Neurodegeneration. Brain.

[47] Pérez V.I., Lew C.M., Cortez L.A., Webb C.R., Rodriguez M., Liu Y., Qi W., Li Y., Chaudhuri A., van Remmen H. (2008). Thioredoxin 2 Haploinsufficiency in Mice Results in Impaired Mitochondrial Function and Increased Oxidative Stress. Free Radic. Biol. Med..

[48] Jiang N., Liu J., Guan C., Ma C., An J., Tang X. (2022). Thioredoxin-Interacting Protein: A New Therapeutic Target in Bone Metabolism Disorders?. Front. Immunol..

[49] Maggio D., Barabani M., Pierandrei M., Polidori M.C., Catani M., Mecocci P., Senin U., Pacifici R., Cherubini A. (2003). Marked Decrease in Plasma Antioxidants in Aged Osteoporotic Women: Results of a Cross-Sectional Study. J. Clin. Endocrinol. Metab..

[50] Aspera-Werz R.H., Ehnert S., Heid D., Zhu S., Chen T., Braun B., Sreekumar V., Arnscheidt C., Nussler A.K. (2018). Nicotine and Cotinine Inhibit Catalase and Glutathione Reductase Activity Contributing to the Impaired Osteogenesis of SCP-1 Cells Exposed to Cigarette Smoke. Oxidative Med. Cell. Longev..

[51] Chang H.B., Javed A., Dai Q., Kappes J.C., Clemens T.L., Darley-Usmar V.M., McDonald J.M., Chen Y. (2008). Oxidative Stress Induces Vascular Calcification through Modulation of the Osteogenic Transcription Factor Runx2 by AKT Signaling. J. Biol. Chem..

[52] Shimoda-Matsubayashi S., Matsumine H., Kobayashi T., Nakagawa-Hattori Y., Shimizu Y., Mizuno Y. (1996). Structural Dimorphism in the Mitochondrial Targeting Sequence in the Human Manganese Superoxide Dismutase Gene. A Predictive Evidence for Conformational Change to Influence Mitochondrial Transport and a Study of Allelic Association in Parkinson’s Disease. Biochem. Biophys. Res. Commun..

[53] Harsløf T., Husted L.B., Nyegaard M., Carstens M., Stenkjær L., Brixen K., Eiken P., Jensen J.E.B., Børglum A.D., Mosekilde L. (2011). Polymorphisms in the ALOX12 Gene and Osteoporosis. Osteoporos. Int..

[54] Zheng Z., Li Y., Jin G., Huang T., Zou M., Duan S. (2020). The Biological Role of Arachidonic Acid 12-Lipoxygenase (ALOX12) in Various Human Diseases. Biomed. Pharmacother..

[55] Deng H.W., Xu F.H., Huang Q.Y., Shen H., Deng H., Conway T., Liu Y.J., Liu Y.Z., Li J.L., Zhang H.T. (2002). A Whole-Genome Linkage Scan Suggests Several Genomic Regions Potentially Containing Quantitative Trait Loci for Osteoporosis. J. Clin. Endocrinol. Metab..

[56] Khan E., Abu-Amer Y. (2003). Activation of Peroxisome Proliferator-Activated Receptor-γ Inhibits Differentiation of Preosteoblasts. J. Lab. Clin. Med..

[57] Akune T., Ohba S., Kamekura S., Yamaguchi M., Chung U., Kubota N., Terauchi Y., Harada Y., Azuma Y., Nakamura K. (2004). PPARgamma Insufficiency Enhances Osteogenesis through Osteoblast Formation from Bone Marrow Progenitors. J. Clin. Investig..

[58] Lv H., Zhen C., Liu J., Yang P., Hu L., Shang P. (2019). Unraveling the Potential Role of Glutathione in Multiple Forms of Cell Death in Cancer Therapy. Oxidative Med. Cell. Longev..

[59] Sharma T., Islam N., Ahmad J., Akhtar N., Beg M. (2015). Correlation between Bone Mineral Density and Oxidative Stress in Postmenopausal Women. Indian J. Endocrinol. Metab..

[60] Ricciardolo F., Nijkamp F., Folkerts G. (2006). Nitric Oxide Synthase (NOS) as Therapeutic Target for Asthma and Chronic Obstructive Pulmonary Disease. Curr. Drug Targets.

[61] Van’t Hof R.J., Ralston S.H. (2001). Nitric Oxide and Bone. Immunology.

[62] Wimalawansa S.J. (2008). Nitric Oxide: Novel Therapy for Osteoporosis. Expert Opin. Pharmacother..

[63] Jin Z., Kho J., Dawson B., Jiang M.M., Chen Y., Ali S., Burrage L.C., Grover M., Palmer D.J., Turner D.L. (2021). Nitric Oxide Modulates Bone Anabolism through Regulation of Osteoblast Glycolysis and Differentiation. J. Clin. Investig..

[64] Liu H., Rosen C.J. (2021). Nitric Oxide and Bone: The Phoenix Rises Again. J. Clin. Investig..

[65] De Bruin A.M., Voermans C., Nolte M.A. (2014). Impact of Interferon-γ on Hematopoiesis. Blood.

[66] Zhao J., Zhao Q., Ning P., Shang K., Liu C., Ni M., Li C., Zhang K., Gao C. (2019). G-CSF Inhibits Growths of Osteoblasts and Osteocytes by Upregulating Nitric Oxide Production in Neutrophils. J. Craniofacial Surg..

[67] Johnson D.L., McAllister T.N., Frangos J.A. (1996). Fluid Flow Stimulates Rapid and Continuous Release of Nitric Oxide in Osteoblasts. Am. J. Physiol..

[68] Engin K.N. (2009). Alpha-Tocopherol: Looking beyond an Antioxidant. Mol. Vis..

[69] Wong S.K., Mohamad N.V., Ibrahim N.I., Chin K.Y., Shuid A.N., Ima-Nirwana S. (2019). The Molecular Mechanism of Vitamin E as a Bone-Protecting Agent: A Review on Current Evidence. Int. J. Mol. Sci..

[70] Melhus H., Michaëlsson K., Holmberg L., Wolk A., Ljunghall S. (1999). Smoking, Antioxidant Vitamins, and the Risk of Hip Fracture. J. Bone Miner. Res..

[71] Vallibhakara S.A.O., Nakpalat K., Sophonsritsuk A., Tantitham C., Vallibhakara O. (2021). Effect of Vitamin E Supplement on Bone Turnover Markers in Postmenopausal Osteopenic Women: A Double-Blind, Randomized, Placebo-Controlled Trial. Nutrients.

[72] Aung L.H.H., Yin R.X., Wu D.F., Wang W., Liu C.W., Pan S.L. (2014). Association of the Variants in the BUD13-ZNF259 Genes and the Risk of Hyperlipidaemia. J. Cell. Mol. Med..

[73] Zhu Y., Zhang D., Zhou D., Li Z., Li Z., Fang L., Yang M., Shan Z., Li H., Chen J. (2017). Susceptibility Loci for Metabolic Syndrome and Metabolic Components Identified in Han Chinese: A Multi-Stage Genome-Wide Association Study. J. Cell. Mol. Med..

[74] Bai W., Kou C., Zhang L., You Y., Yu W., Hua W., Li Y., Yu Y., Zhao T., Wu Y. (2019). Functional Polymorphisms of the APOA1/C3/A4/A5-ZPR1-BUD13 Gene Cluster Are Associated with Dyslipidemia in a Sex-Specific Pattern. PeerJ.

[75] Major J.M., Yu K., Wheeler W., Zhang H., Cornelis M.C., Wright M.E., Yeager M., Snyder K., Weinstein S.J., Mondul A. (2011). Genome-Wide Association Study Identifies Common Variants Associated with Circulating Vitamin E Levels. Hum. Mol. Genet..

[76] Bartolini D., Marinelli R., Giusepponi D., Galarini R., Barola C., Stabile A.M., Sebastiani B., Paoletti F., Betti M., Rende M. (2021). Alpha-Tocopherol Metabolites (the Vitamin E Metabolome) and Their Interindividual Variability during Supplementation. Antioxidants.

[77] Brodeur M.R., Brissette L., Falstrault L., Luangrath V., Moreau R. (2008). Scavenger Receptor of Class B Expressed by Osteoblastic Cells Are Implicated in the Uptake of Cholesteryl Ester and Estradiol from LDL and HDL3. J. Bone Miner. Res..

[78] Martineau C., Martin-Falstrault L., Brissette L., Moreau R. (2014). The Atherogenic Scarb1 Null Mouse Model Shows a High Bone Mass Phenotype. Am. J. Physiol. Endocrinol. Metab..

[79] Lacombe J., Karsenty G., Ferron M. (2013). Regulation of Lysosome Biogenesis and Functions in Osteoclasts. Cell Cycle.

[80] Gary-Bobo M., Nirde P., Jeanjean A., Morere A., Garcia M. (2007). Mannose 6-Phosphate Receptor Targeting and Its Applications in Human Diseases. Curr. Med. Chem..

[81] Dahms N.M., Olson L.J., Kim J.J.P. (2008). Strategies for Carbohydrate Recognition by the Mannose 6-Phosphate Receptors. Glycobiology.

[82] Kollmann K., Pestka J.M., Kühn S.C., Schöne E., Schweizer M., Karkmann K., Otomo T., Catala-Lehnen P., Failla A.V., Marshall R.P. (2013). Decreased Bone Formation and Increased Osteoclastogenesis Cause Bone Loss in Mucolipidosis II. EMBO Mol. Med..

[83] Koehne T., Markmann S., Schweizer M., Muschol N., Friedrich R.E., Hagel C., Glatzel M., Kahl-Nieke B., Amling M., Schinke T. (2016). Mannose 6-Phosphate-Dependent Targeting of Lysosomal Enzymes Is Required for Normal Craniofacial and Dental Development. Biochim. Biophys. Acta.

