# Supplementary material for: Oxidative-Stress-Related Genes in Osteoporosis: A Systematic Review
Source: Antioxidants (Basel). 2023 Apr 12;12(4):915. doi: 10.3390/antiox12040915 (PMC10135393; doi:10.3390/antiox12040915)
Supplement: Supplementary file 1 [file antioxidants-12-00915-s001.zip › antioxidants-2299451-supplementary.pdf]

Table S1. Search strategy in the website database.

Data base	Search strategy	Date
PubMed	((gene) OR (single nucleotide polymorphism) OR (genetic variant)) AND ((oxidative stress) OR (antioxidant)) AND ((osteoporosis) OR (bone mineral density) OR (fracture)) AND (adults)	November 1st, 2022.
WEB OF SCIENCE	((gene) OR (single nucleotide polymorphism) OR (genetic variant)) AND ((oxidative stress) OR (antioxidant)) AND ((osteoporosis) OR (bone mineral density) OR (fracture)) AND (adults)	November 1st, 2022.
EBSCO	AB oxidative stress AND AB genes AND AB (osteoporosis or bone density or bone loss)	November 1st, 2022.
SCOPUS	((gene) OR (single nucleotide polymorphism) OR (genetic variant)) AND ((oxidative stress) OR (antioxidant)) AND ((osteoporosis) OR (bone mineral density) OR (fracture)) AND (adults)	November 1st, 2022.
BVS	((gene) OR (single nucleotide polymorphism) OR (genetic variant)) AND ((oxidative stress) OR (antioxidant)) AND ((osteoporosis) OR (bone mineral density) OR (fracture)) AND (adults)	November 1st, 2022.

Table S2. Excluded articles.

Country	Reference	Population	Outcome	Exposure	Language	Others
Australia	Brennan-Olsen SL, Page RS, Berk M, et al. DNA methylation and the social gradient of osteoporotic fracture: A conceptual model. BONE. 2016;84:204-212. doi:10.1016/j.bone.2015.12.015			epigenetics		review
Austria	Anour R, Andrukhova O, Ritter E, Zeitz U, Erben RG. Klotho Lacks a Vitamin D Independent Physiological Role in Glucose Homeostasis, Bone Turnover, and Steady-State PTH Secretion In Vivo. PLoS ONE. 2012;7(2):1-10. doi:10.1371/journal.pone.0031376	animals		administration assay		
Austria	Föger-Samwald U, Vekszler G, Hörz-Schuch E, Salem S, Wipperich M, Ritschl P, Mousavi M, Pietschmann P. Molecular mechanisms of osteoporotic hip fractures in elderly women. Exp Gerontol. 2016 Jan;73:49-58. doi: 10.1016/j.exger.2015.11.012. Epub 2015 Dec 1. PMID: 26608808.	cell culture		transcriptomics		
Austria	Gruber, R., et al. "Fracture Healing in the Elderly Patient." Experimental Gerontology, vol. 41, no. 11, 2006, pp. 1080-1093. SCOPUS, www.scopus.com, doi:10.1016/j.exger.2006.09.008.	cell culture				review
Austria	Pietschmann P, Mechtcheriakova D, Meshcheryakova A, Föger-Samwald U, Ellinger I. Immunology of Osteoporosis: A Mini-Review. Gerontology. 2016;62(2):128-137. doi:10.1159/000431091					review
Austria	Stähli A, Ubaidha Maheen C, Josef Strauss F, Eick S, Sculean A, Gruber R. Caffeic acid phenethyl ester protects from oxidative stress and dampens inflammation via heme oxygenase 1. Clinical Oral Implants Research. 2018;29(17):253. doi:10.1111/clr.138_13358	cell culture	periodontitis			
Austria	Zupan, J., et al. "Age-Related Alterations and Senescence of Mesenchymal Stromal Cells: Implications for Regenerative Treatments of Bones and Joints." Mechanisms of Ageing and Development, vol. 198, 2021. SCOPUS, www.scopus.com, doi:10.1016/j.mad.2021.111539.	cell culture				review
Belgium	Dontaine, P., et al. "Digestive Involvement in a Severe Form of Snyder-Robinson Syndrome: Possible Expansion of the Phenotype." European Journal of Medical Genetics, vol. 64, no. 1, 2021. SCOPUS, www.scopus.com, doi:10.1016/j.ejmg.2020.104097.		Snyder-Robinson syndrome			
Belgium	Vo, T. K. D., et al. "Differentially Abundant Transcripts in PBMC of Hospitalized Geriatric Patients with Hip Fracture Compared to Healthy Aged Controls." Experimental Gerontology, vol. 46, no. 4, 2011, pp. 257-264. SCOPUS, www.scopus.com, doi:10.1016/j.exger.2010.10.012.	cell culture		transcriptomics		
Belgium	Vo, T. K. D., et al. "Transcriptomic Biomarkers of the Response of Hospitalized Geriatric Patients Admitted with Heart Failure. Comparison to Hospitalized Geriatric Patients with Infectious Diseases Or Hip Fracture." Mechanisms of Ageing and Development, vol. 132, no. 3, 2011, pp. 131-139. SCOPUS, www.scopus.com, doi:10.1016/j.mad.2011.02.002.			transcriptomics		
Brazil	Araújo AA de, Pereira A de SBF, Medeiros CACX de, et al. Effects of metformin on inflammation, oxidative stress, and bone loss in a rat model of periodontitis. PLoS ONE. 2017;12(8):1-21. doi:10.1371/journal.pone.0183506	animals	periodontitis	administration assay		
Brazil	Barakat B, Almeida MEF. Biochemical and immunological changes in obesity. Archives of Biochemistry & Biophysics. 2021;708:N.PAG. doi:10.1016/j.abb.2021.108951		obesity			review

Brazil	Fernandes-Breitenbach F, Peres-Ueno MJ, Santos LFG, et al. Analysis of the femoral neck from rats in the periestropause treated with oxytocin and submitted to strength training. BONE. 2022;162:N.PAG. doi:10.1016/j.bone.2022.116452	animals		functional assay	
Brazil	Freire JMO, Chaves HV, Teixeira AH, et al. Protective effect of Platymiscium floribundum Vog. in tree extract on periodontitis inflammation in rats. PLoS ONE. 2019;14(11):1-15. doi:10.1371/journal.pone.0223800	animals	periodontitis	administration assay	
Brazil	Marson, F. A. D. L., et al. "Polymorphisms in the Glutathione Pathway Modulate Cystic Fibrosis Severity: A Cross-Sectional Study." BMC Medical Genetics, vol. 15, no. 1, 2014. SCOPUS, www.scopus.com, doi:10.1186/1471-2350-15-27.		Cystic fibrosis		
Brazil	Martins CS, Leitão RFC, Costa DVS, et al. Topical HPMC/S-Nitrosoglutathione Solution Decreases Inflammation and Bone Resorption in Experimental Periodontal Disease in Rats. PLoS ONE. 2016;11(4):1-19. doi:10.1371/journal.pone.0153716	animals	periodontitis	administration assay	
Brazil	Oliveira GR, Vargas-Sanchez PK, Fernandes RR, et al. Lycopene influences osteoblast functional activity and prevents femur bone loss in female rats submitted to an experimental model of osteoporosis. Journal of Bone & Mineral Metabolism. 2019;37(4):658-667. doi:10.1007/s00774-018-0970-8	animals		administration assay	
Brazil	Souza Marinho D, Longoni Calió M, Mi Ko G, Bertoncini CRA. 223 - Evaluation Effects of Estrogen and Isoflavones of Soy on Oxidative Stress in the Adrenal Glands of Rats. Free Radical Biology & Medicine. 2016;100:S104. doi:10.1016/j.freeradbiomed.2016.10.264	animals		functional assay	
Canada	Blewett TA, Delompré PL, He Y, Folkerts EJ, Flynn SL, Alessi DS, Goss GG. Sublethal and Reproductive Effects of Acute and Chronic Exposure to Flowback and Produced Water from Hydraulic Fracturing on the Water Flea Daphnia magna. Environ Sci Technol. 2017 Mar 7;51(5):3032-3039. doi: 10.1021/acs.est.6b05179. Epub 2017 Feb 13. PMID: 28140571.	animals		functional assay	
Canada	Cui, Q. -., et al. "Sublethal Oligodendrocyte Injury: A Reversible Condition in Multiple Sclerosis?" Annals of Neurology, vol. 81, no. 6, 2017, pp. 811-824. SCOPUS, www.scopus.com, doi:10.1002/ana.24944.	cell culture			
Canada	Da Costa LA, Badawi A, El-Sohemy A. Nutrigenetics and Modulation of Oxidative Stress. Annals of Nutrition & Metabolism. 2012;60:27-36. doi:10.1159/000337311				review
Canada	Kilby, K., et al. "Micronutrient Absorption and Related Outcomes in People with Inflammatory Bowel Disease: A Review." Nutrients, vol. 11, no. 6, 2019. SCOPUS, www.scopus.com, doi:10.3390/nu11061388.				review
Canada	Sivasubramaniyam T, Yang J, Cheng HS, Zyla A, Li A, Besla R, Dotan I, Revelo XS, Shi SY, Le H, Schroer SA, Dodington DW, Park YJ, Kim MJ, Febbraro D, Ruel I, Genest J, Kim RH, Mak TW, Winer DA, Robbins CS, Woo M. Dj1 deficiency protects against atherosclerosis with anti-inflammatory response in macrophages. Sci Rep. 2021 Feb 25;11(1):4723. doi: 10.1038/s41598-021-84063-6. PMID: 33633277; PMCID: PMC7907332.	animals	atherosclerosis		
Chile	Román, F., et al. "Real-Time H2O2 Measurements in Bone Marrow Mesenchymal Stem Cells (MSCs) show Increased Antioxidant Capacity in Cells from Osteoporotic Women." Journal of Cellular Biochemistry, vol. 118, no. 3, 2017, pp. 585-593. SCOPUS, www.scopus.com, doi:10.1002/jcb.25739.	cell culture		administration assay	

China	Chen B, Yan Y-L, Liu C, et al. Therapeutic Effect of Deferoxamine on Iron Overload-Induced Inhibition of Osteogenesis in a Zebrafish Model. Calcified Tissue International. 2014;94(3):353-360. doi:10.1007/s00223-013-9817-4	animals		administration assay	
China	Chen C, Xu C, Zhou T, Gao B, Zhou H, Chen C, Zhang C, Huang D, Su P. Abnormal osteogenic and chondrogenic differentiation of human mesenchymal stem cells from patients with adolescent idiopathic scoliosis in response to melatonin. Mol Med Rep. 2016 Aug;14(2):1201-9. doi: 10.3892/mmr.2016.5384. Epub 2016 Jun 10. PMID: 27314307; PMCID: PMC4940077.	cell culture		administration assay	
China	Chen W, Chen X, Chen AC, et al. Melatonin restores the osteoporosis-impaired osteogenic potential of bone marrow mesenchymal stem cells by preserving SIRT1-mediated intracellular antioxidant properties. Free Radical Biology & Medicine. 2020;146:92-106. doi:10.1016/j.freeradbiomed.2019.10.412	animals		administration assay	
China	Chen X, Wang C, Qiu H, Yuan Y, Chen K, Cao Z, Xiang Tan R, Tickner J, Xu J, Zou J. Asperpyrone A attenuates RANKL-induced osteoclast formation through inhibiting NFATc1, Ca2+ signalling and oxidative stress. J Cell Mol Med. 2019 Dec;23(12):8269-8279. doi: 10.1111/jcmm.14700. Epub 2019 Oct 15. PMID: 31612613; PMCID: PMC6850946.	cell culture	adiposity	administration assay	
China	Chen X, Zhu X, Wei A, Chen F, Gao Q, Lu K, Jiang Q, Cao W. Nrf2 epigenetic derepression induced by running exercise protects against osteoporosis. Bone Res. 2021 Feb 26;9(1):15. doi: 10.1038/s41413-020-00128-8. PMID: 33637693; PMCID: PMC7910611.	animals		epigenetics	
China	Chen, J. -., et al. "Mito-TEMPO Attenuates Oxidative Stress and Mitochondrial Dysfunction in Noise-Induced Hearing Loss Via Maintaining TFAM-mtDNA Interaction and Mitochondrial Biogenesis." Frontiers in Cellular Neuroscience, vol. 16, 2022. SCOPUS, www.scopus.com, doi:10.3389/fncel.2022.803718.	animals		mitochondria	
China	Chen, J., et al. "Exploring the Temporal Correlation of Sarcopenia with Bone Mineral Density and the Effects of Osteoblast-Derived Exosomes on Myoblasts through an Oxidative Stress-Related Gene." Oxidative Medicine and Cellular Longevity, vol. 2022, 2022. SCOPUS, www.scopus.com, doi:10.1155/2022/9774570.	animals		transcriptomics ; administration assay	
China	Chen, M., Y. Li, and G. Huang. "Potential Health Functions of Collagen Bioactive Peptides: A Review." American Journal of Biochemistry and Biotechnology, vol. 16, no. 4, 2020, pp. 507-519. SCOPUS, www.scopus.com, doi:10.3844/ajbbsp.2020.507.519.				review
China	Cong, L., et al. "Upregulation of FoxO6 in Nucleus Pulposus Cells Promotes DNA Damage Repair Via Activation of RAD51." European Review for Medical and Pharmacological Sciences, vol. 25, no. 17, 2021, pp. 5392-5401. SCOPUS, www.scopus.com, doi:10.26355/eurrev_202109_26646.	cell culture		functional assay	
China	Deng FY, Liu YZ, Li LM, Jiang C, Wu S, Chen Y, Jiang H, Yang F, Xiong JX, Xiao P, Xiao SM, Tan LJ, Sun X, Zhu XZ, Liu MY, Lei SF, Chen XD, Xie JY, Xiao GG, Liang SP, Deng HW. Proteomic analysis of circulating monocytes in Chinese premenopausal females with extremely discordant bone mineral density. Proteomics. 2008 Oct;8(20):4259-72. doi: 10.1002/pmic.200700480. PMID: 18924182; PMCID: PMC2760933.	cell culture		proteomics	
China	Feng J, Liu S, Ma S, Zhao J, Zhang W, Qi W, Cao P, Wang Z, Lei W. Protective effects of resveratrol on postmenopausal osteoporosis: regulation of SIRT1-NF-κB signaling pathway. Acta Biochim Biophys Sin (Shanghai). 2014 Dec;46(12):1024-33. doi: 10.1093/abbs/gmu103. Epub 2014 Nov 6. PMID: 25377437.	animals	periodontitis	administration assay	

China	Fu C, Xu D, Wang C, et al. Alpha-Lipoic Acid Promotes Osteoblastic Formation in H2O2-Treated MC3T3-E1 Cells and Prevents Bone Loss in Ovariectomized Rats. Journal of Cellular Physiology. 2015;230(9):2184-2201. doi:10.1002/jcp.24947	animals		functional assay	
China	Gu, Z., et al. "MicroRNA-497 Elevation Or LRG1 Knockdown Promotes Osteoblast Proliferation and Collagen Synthesis in Osteoporosis Via TGF-β1/Smads Signalling Pathway." Journal of Cellular and Molecular Medicine, vol. 24, no. 21, 2020, pp. 12619-12632. SCOPUS, www.scopus.com, doi:10.1111/jcmm.15826.	animals		transcriptomics ; functional assay	
China	Guoju Hong, Lin Zhou, Xuguang Shi, et al. Bajijiasu Abrogates Osteoclast Differentiation via the Suppression of RANKL Signaling Pathways through NF-κB and NFAT. International Journal of Molecular Sciences. 2017;18(1):203. doi:10.3390/ijms18010203	cell culture		functional assay	
China	He Y, Zhang L, Zhu Z, Xiao A, Yu H, Gan X. Blockade of cyclophilin D rescues dexamethasone-induced oxidative stress in gingival tissue. PLoS ONE. 2017;12(3):1-13. doi:10.1371/journal.pone.0173270	cell culture	gingival injury	functional assay	
China	Hu, X., et al. "GPX7 Facilitates BMSCs Osteoblastogenesis Via ER Stress and mTOR Pathway." Journal of Cellular and Molecular Medicine, vol. 25, no. 22, 2021, pp. 10454-10465. SCOPUS, www.scopus.com, doi:10.1111/jcmm.16974.	cell culture		functional assay	
China	Huang J, Ye Z, Wang J, Chen Q, Huang D, Liu H. USP13 mediates PTEN to ameliorate osteoarthritis by restraining oxidative stress, apoptosis and inflammation via AKT-dependent manner. Biomedicine & Pharmacotherapy. 2021;133:N.PAG. doi:10.1016/j.biopha.2020.111089	animals		transcriptomics	
China	Huang Q, Shi J, Gao B, et al. Gastrodin: An ancient Chinese herbal medicine as a source for anti-osteoporosis agents via reducing reactive oxygen species. BONE. 2015;73:132-144. doi:10.1016/j.bone.2014.12.059	cell culture		administration assay	
China	Huang Y, Zhai X, Ma T, et al. Rare earth-based materials for bone regeneration: Breakthroughs and advantages. Coordination Chemistry Reviews. 2022;450:N.PAG. doi:10.1016/j.ccr.2021.214236				review
China	Huang, Q., et al. "Protective Effects of Myricitrin Against Osteoporosis Via Reducing Reactive Oxygen Species and Bone-Resorbing Cytokines." Toxicology and Applied Pharmacology, vol. 280, no. 3, 2014, pp. 550-560. SCOPUS, www.scopus.com, doi:10.1016/j.taap.2014.08.004.	animals		functional assay	
China	Jiang, X., et al. "Key LncRNAs Associated with Oxidative Stress were Identified by GEO Database Data and Whole Blood Analysis of Intervertebral Disc Degeneration Patients." Frontiers in Genetics, vol. 13, 2022. SCOPUS, www.scopus.com, doi:10.3389/fgene.2022.929843.		Intervertebral disc degeneration (IDD	transcriptomics	
China	Kong Y, Nie Z-K, Li F, Guo H-M, Yang X-L, Ding S-F. MiR-320a was highly expressed in postmenopausal osteoporosis and acts as a negative regulator in MC3T3E1 cells by reducing MAP9 and inhibiting PI3K/AKT signaling pathway. Experimental & Molecular Pathology. 2019;110:N.PAG. doi:10.1016/j.yexmp.2019.104282	cell culture		transcriptomics	
China	Lan C, Long L, Xie K, et al. miRNA-429 suppresses osteogenic differentiation of human adipose-derived mesenchymal stem cells under oxidative stress via targeting SCD-1. Experimental & Therapeutic Medicine. 2020;19(1):696-702. doi:10.3892/etm.2019.8246	cell culture		transcriptomics	

China	Li C, Zhang J, Lv F, Ge X, Li G. Naringin protects against bone loss in steroid-treated inflammatory bowel disease in a rat model. Archives of Biochemistry & Biophysics. 2018;650:22-29. doi:10.1016/j.abb.2018.05.011	animals	administration assay
China	Li J, Wang Q, Yang R, et al. BMI-1 Mediates Estrogen-Deficiency-Induced Bone Loss by Inhibiting Reactive Oxygen Species Accumulation and T Cell Activation. Journal of Bone & Mineral Research. 2017;32(5):962-973. Accessed November 5, 2022. https://search.ebscohost.com/login.aspx?direct=true&AuthType=sso&db=s3h&AN=122812490&lang=es&site=ehost-live	animals	functional assay
China	Li S, Gao L, Zhang W, et al. MiR-152-5p suppresses osteogenic differentiation of mandible mesenchymal stem cells by regulating ATG14-mediated autophagy. Stem Cell Research & Therapy. 2022;13(1):1-17. doi:10.1186/s13287-022-03018-4	cell culture	transcriptomics
China	Li Y, Tan Y, Zhang G, Yang B, Zhang J. Effects of calcitonin gene-related peptide on the expression and activity of nitric oxide synthase during mandibular bone healing in rabbits: an experimental study. J Oral Maxillofac Surg. 2009 Feb;67(2):273-9. doi: 10.1016/j.joms.2008.06.077. PMID: 19138599.	animals	functional assay
China	Li Z, Chen C, Zhu X, Li Y, Yu R, Xu W. Glycyrrhizin Suppresses RANKL-Induced Osteoclastogenesis and Oxidative Stress Through Inhibiting NF-κB and MAPK and Activating AMPK/Nrf2. Calcified Tissue International. 2018;103(3):324-337. doi:10.1007/s00223-018-0425-1	cell culture	administration assay
China	Li, L., et al. "Oxidative Stress-Induced Hypermethylation of KLF5 Promoter Mediated by DNMT3B Impairs Osteogenesis by Diminishing the Interaction with β-Catenin." Antioxidants and Redox Signaling, vol. 35, no. 1, 2021, pp. 1-20. SCOPUS, www.scopus.com, doi:10.1089/ars.2020.8200.	cell culture	functional assay
China	Liu G, Zhou H, Li Y, Li G, Cui L, Liu W, Cao Y. Evaluation of the viability and osteogenic differentiation of cryopreserved human adipose-derived stem cells. Cryobiology. 2008 Aug;57(1):18-24. doi: 10.1016/j.cryobiol.2008.04.002. Epub 2008 May 21. PMID: 18495102.	cell culture	
China	Liu H, Bian W, Liu S, Huang K. Selenium protects bone marrow stromal cells against hydrogen peroxide-induced inhibition of osteoblastic differentiation by suppressing oxidative stress and ERK signaling pathway. Biol Trace Elem Res. 2012 Dec;150(1-3):441-50. doi: 10.1007/s12011-012-9488-4. Epub 2012 Aug 15. PMID: 22890880.	animals	functional assay
China	Lu T, Parthasarathy S, Hao H, Luo M, Ahmed S, Zhu J, Luo S, Kuppusamy P, Sen CK, Verfaillie CM, Tian J, Liu Z. Reactive oxygen species mediate oxidized low-density lipoprotein-induced inhibition of oct-4 expression and endothelial differentiation of bone marrow stem cells. Antioxid Redox Signal. 2010 Dec 15;13(12):1845-56. doi: 10.1089/ars.2010.3156. Epub 2010 Oct 12. PMID: 20836655; PMCID: PMC2971633.	animals	functional assay
China	Lv H, Che T, Tang X, Liu L, Cheng J. Puerarin enhances proliferation and osteoblastic differentiation of human bone marrow stromal cells via a nitric oxide/cyclic guanosine monophosphate signaling pathway. Mol Med Rep. 2015 Aug;12(2):2283-90. doi: 10.3892/mmr.2015.3647. Epub 2015 Apr 20. PMID: 25892538.	cell culture	administration assay
China	Lv H, Ma X, Che T, Chen Y. Methylation of the promoter A of estrogen receptor alpha gene in hBMSC and osteoblasts and its correlation with homocysteine. Mol Cell Biochem. 2011 Sep;355(1-2):35-45. doi: 10.1007/s11010-011-0836-z. Epub 2011 Apr 27. PMID: 21523370.		epigenetics

China	Ma H -P., Ma X -N., Ge B -F., et al. Icariin attenuates hypoxia-induced oxidative stress and apoptosis in osteoblasts and preserves their osteogenic differentiation potential in vitro. Cell Proliferation. 2014;47(6):527-539. doi:10.1111/cpr.12147	animals		administration assay
China	Man GC, Wang WW, Yeung BH, Lee SK, Ng BK, Hung WY, Wong JH, Ng TB, Qiu Y, Cheng JC. Abnormal proliferation and differentiation of osteoblasts from girls with adolescent idiopathic scoliosis to melatonin. J Pineal Res. 2010 Aug;49(1):69-77. doi: 10.1111/j.1600-079X.2010.00768.x. Epub 2010 May 27. PMID: 20524972.	cell culture		administration assay
China	Mao W, Zhu Z. Parthenolide inhibits hydrogen peroxide-induced osteoblast apoptosis. Molecular Medicine Reports. 2018;17(6):8369-8376. doi:10.3892/mmr.2018.8908	cell culture		administration assay
China	Meng J, Zhang X, Guo X, Cheng W, Qi X, Huang J, Lin W. Briarane-type diterpenoids suppress osteoclastogenisis by regulation of Nrf2 and MAPK/NF-kB signaling pathway. Bioorg Chem. 2021 Jul;112:104976. doi: 10.1016/j.bioorg.2021.104976. Epub 2021 May 7. PMID: 33992967.	cell culture		administration assay
China	Pang C, Wen L, Lu X, et al. Ruboxistaurin maintains the bone mass of subchondral bone for blunting osteoarthritis progression by inhibition of osteoclastogenesis and bone resorption activity. Biomedicine & Pharmacotherapy. 2020;131:N.PAG. doi:10.1016/j.biopha.2020.110650	cell culture		administration assay
China	Peng M, Qiang L, Xu Y, Li C, Li T, Wang J. Inhibition of JNK and activation of the AMPK-Nrf2 axis by corosolic acid suppress osteolysis and oxidative stress. Nitric Oxide. 2019;82:12-24. doi:10.1016/j.niox.2018.11.002	animals		administration assay
China	Shen J, Hu Z, Zhong X, Wang D, Xu L. [Restoring phenotype of dedifferentiated normal nucleus pulposus cells by resveratrol]. Zhongguo Xiu Fu Chong Jian Wai Ke Za Zhi. 2013 May;27(5):547-53. Chinese. PMID: 23879090.	cell culture		functional assay
China	Sun H, Qiao W, Cui M, et al. The Polycomb Protein Bmi1 Plays a Crucial Role in the Prevention of 1,25(OH)2D Deficiency-Induced Bone Loss. Journal of Bone & Mineral Research. 2020;35(3):583-595. Accessed November 5, 2022. https://search.ebscohost.com/login.aspx?direct=true&AuthType=sso&db=s3h&AN=142137224&lang=es&site=ehost-live	animals		administration assay
China	Sun X, Mao Y, Dai P, et al. Mitochondrial dysfunction is involved in the aggravation of periodontitis by diabetes. Journal of Clinical Periodontology. 2017;44(5):463-471. doi:10.1111/jcpe.12711	animals	periodontitis	mitochondria
China	Sun X, Xia T, Zhang S, et al. Hops extract and xanthohumol ameliorate bone loss induced by iron overload via activating Akt/GSK3β/Nrf2 pathway. Journal of Bone & Mineral Metabolism. 2022;40(3):375-388. doi:10.1007/s00774-021-01295-2	animals		administration assay
China	Sun X, Yuan Y, Xiao Y, et al. Long non-coding RNA, Bmcob, regulates osteoblastic differentiation of bone marrow mesenchymal stem cells. Biochemical & Biophysical Research Communications. 2018;506(3):536-542. doi:10.1016/j.bbrc.2018.09.142	cell culture		transcriptomics
China	Sun Z, Yang S, Ye S, Zhang Y, Xu W, Zhang B, Liu X, Mo F, Hua W. Aberrant CpG islands' hypermethylation of ABCB1 in mesenchymal stem cells of patients with steroid-associated osteonecrosis. J Rheumatol. 2013 Nov;40(11):1913-20. doi: 10.3899/jrheum.130191. Epub 2013 Sep 15. PMID: 24037553.	cell culture		epigenetics
China	Sun ZB, Wang JW, Xiao H, Zhang QS, Kan WS, Mo FB, Hu S, Ye SN. Icariin may benefit the mesenchymal stem cells of patients with steroid-associated osteonecrosis by ABCB1-promoter	cell culture		administration assay

	demethylation: a preliminary study. Osteoporos Int. 2015 Jan;26(1):187-97. doi: 10.1007/s00198-014-2809-z. Epub 2014 Aug 12. PMID: 25112719.		
China	Su-Qin Zhang, Wai-Jiao Cai, Jian-Hua Huang, et al. Icariin, a natural flavonol glycoside, extends healthspan in mice. Experimental Gerontology. 2015;69:226-235. doi:10.1016/j.exger.2015.06.020	animals	administration assay
China	Tan J, Xu X, Tong Z, Lin J, Yu Q, Lin Y, Kuang W. Decreased osteogenesis of adult mesenchymal stem cells by reactive oxygen species under cyclic stretch: a possible mechanism of age related osteoporosis. Bone Res. 2015 Mar 17;3:15003. doi: 10.1038/boneres.2015.3. PMID: 26273536; PMCID: PMC4413016.	cell culture	functional assay
China	Tao Z-S, Li T-L, Wei S. Silymarin prevents iron overload induced bone loss by inhibiting oxidative stress in an ovariectomized animal model. Chemico-Biological Interactions. 2022;366:N.PAG. doi:10.1016/j.cbi.2022.110168	animals	administration assay
China	Terruzzi I, Montesano A, Senesi P, Villa I, Ferraretto A, Bottani M, Vacante F, Spinello A, Bolamperti S, Luzi L, Rubinacci A. L-Carnitine Reduces Oxidative Stress and Promotes Cells Differentiation and Bone Matrix Proteins Expression in Human Osteoblast-Like Cells. Biomed Res Int. 2019 Jan 20;2019:5678548. doi: 10.1155/2019/5678548. PMID: 30800672; PMCID: PMC6360619.	animals	administration assay
China	Wang D, Hu Z, Hao J, He B, Gan Q, Zhong X, Zhang X, Shen J, Fang J, Jiang W. SIRT1 inhibits apoptosis of degenerative human disc nucleus pulposus cells through activation of Akt pathway. Age (Dordr). 2013 Oct;35(5):1741-53. doi: 10.1007/s11357-012-9474-y. Epub 2012 Sep 19. PMID: 22990594; PMCID: PMC3776108.	cell culture	functional assay
China	Wang F, Wu X, Wang Y, Li G, Zhang M. [An in vitro study on human bone marrow mesenchymal stem cells protecting nucleus pulposus cells from oxidative stress-induced apoptosis in a co-culture system of no direct cellular interaction]. Zhongguo Xiu Fu Chong Jian Wai Ke Za Zhi. 2010 Apr;24(4):391-8. Chinese. PMID: 20458997.	cell culture	functional assay
China	Wang K, Zhou C, Li L, et al. Aucubin promotes bone-fracture healing via the dual effects of anti-oxidative damage and enhancing osteoblastogenesis of hBM-MSCs. Stem Cell Research & Therapy. 2022;13(1):1-13. doi:10.1186/s13287-022-03125-2	animals; cell culture	functional assay
China	Wang L, Zhang Y-G, Wang X-M, Ma L-F, Zhang Y-M. Naringin protects human adipose-derived mesenchymal stem cells against hydrogen peroxide-induced inhibition of osteogenic differentiation. Chemico-Biological Interactions. 2015;242:255-261. doi:10.1016/j.cbi.2015.10.010	cell culture	administration assay
China	Wang X, Tang S, Chai S, Wang P, Qin J, Pei W, Bian H, Jiang Q, Huang C. Preparing printable bacterial cellulose based gelatin gel to promote in vivo bone regeneration. Carbohydr Polym. 2021 Oct 15;270:118342. doi: 10.1016/j.carbpol.2021.118342. Epub 2021 Jun 21. PMID: 34364595.	cell culture	administration assay
China	Wang, N., et al. "Quercetin Promotes Osteogenic Differentiation and Antioxidant Responses of Mouse Bone Mesenchymal Stem Cells through Activation of the AMPK/SIRT1 Signaling Pathway." Phytotherapy Research, vol. 35, no. 5, 2021, pp. 2639-2650. SCOPUS, www.scopus.com, doi:10.1002/ptr.7010.	animals	administration assay

China	Wei L, Chen W, Huang L, et al. Alpinetin ameliorates bone loss in LPS-induced inflammation osteolysis via ROS mediated P38/PI3K signaling pathway. Pharmacological Research. 2022;184:N.PAG. doi:10.1016/j.phrs.2022.106400	animals		functional assay	
China	Wu JW, Wang JJ, Chen JB, Huang YL, Wang H, Liu GH, Li LF, Kang M, Wang XG, Cai HH. Resveratrol could reverse the expression of SIRT1 and MMP-1 in vitro. Genet Mol Res. 2015 Oct 16;14(4):12386-93. doi: 10.4238/2015.October.16.5. PMID: 26505388.	cell culture		administration assay	
China	Xiao Y, Cui J, Shi Y, Sun J, Wang Z, Le G. Effects of duodenal redox status on calcium absorption and related genes expression in high-fat diet–fed mice. Nutrition. 2010;26(11/12):1188-1194. doi:10.1016/j.nut.2009.11.021	animals		transcriptomics ; diet intervention	
China	Xiao, J., et al. "STK11 Overexpression Prevents Glucocorticoid-Induced Osteoporosis Via Activating the AMPK/SIRT1/PGC1α Axis." Human Cell, vol. 35, no. 4, 2022, pp. 1045-1059. SCOPUS, www.scopus.com, doi:10.1007/s13577-022-00704-6.	cell culture		transcriptomics ; functional assay	
China	Xie Y, Gao Y, Zhang L, Chen Y, Ge W, Tang P. Involvement of serum-derived exosomes of elderly patients with bone loss in failure of bone remodeling via alteration of exosomal bone-related proteins. Aging Cell. 2018 Jun;17(3):e12758. doi: 10.1111/acel.12758. Epub 2018 Mar 30. PMID: 29603567; PMCID: PMC5946082.			exomes	
China	Xie Y, Han N, Li F, Wang L, Liu G, Hu M, Wang S, Wei X, Guo J, Jiang H, Wang J, Li X, Wang Y, Wang J, Bian X, Zhu Z, Zhang H, Liu C, Liu X, Liu Z. Melatonin enhances osteoblastogenesis of senescent bone marrow stromal cells through NSD2-mediated chromatin remodelling. Clin Transl Med. 2022 Feb;12(2):e746. doi: 10.1002/ctm2.746. PMID: 35220680; PMCID: PMC8882236.	cell culture		administration assay	
China	Xiong A, Yao Q, He J, Fu W, Yu J, Zhang Z. No causal effect of serum urate on bone-related outcomes among a population of postmenopausal women and elderly men of Chinese Han ethnicity--a Mendelian randomization study. Osteoporos Int. 2016 Mar;27(3):1031-1039. doi: 10.1007/s00198-015-3341-5. Epub 2015 Nov 20. PMID: 26588908.			Mendelian randomization analysis	
China	Xiong Y, Zhang Y, Zhou F, et al. FOXO1 differentially regulates bone formation in young and aged mice. Cellular Signalling. 2022;99:N.PAG. doi:10.1016/j.cellsig.2022.110438	animals			
China	Yang K, Li J, Tao L. Purine metabolism in the development of osteoporosis. Biomedicine & Pharmacotherapy. 2022;155:N.PAG. doi:10.1016/j.biopha.2022.113784		purine metabolism		review
China	Yang P, Feng Q, Meng L, Tang R, Jiang Y, Liu H, Si H, Li M. The mechanism underlying the TC-G 1008 rescue of reactive oxygen species (ROS)-induced osteoblast apoptosis by the upregulation of peroxiredoxin 1. Int J Biochem Cell Biol. 2022 Oct;151:106276. doi: 10.1016/j.biocel.2022.106276. Epub 2022 Aug 8. PMID: 35953014.	cell culture		functional assay	
China	Yang, X., et al. "Changes in the Composition of Gut and Vaginal Microbiota in Patients with Postmenopausal Osteoporosis." Frontiers in Immunology, vol. 13, 2022. SCOPUS, www.scopus.com, doi:10.3389/fimmu.2022.930244.			mitochondria	
China	Yang, Y., et al. "Oxidative Stress Induces Downregulation of TP53INP2 and Suppresses Osteogenic Differentiation of BMSCs during Osteoporosis through the Autophagy Degradation Pathway." Free Radical Biology and Medicine, vol. 166, 2021, pp. 226-237. SCOPUS, www.scopus.com, doi:10.1016/j.freeradbiomed.2021.02.025.	cell culture		functional assay	

China	Yao H, Yao Z, Zhang S, Zhang Wen, Zhou W. Upregulation of SIRT1 inhibits H2O2-induced osteoblast apoptosis via FoxO1/β-catenin pathway. Molecular Medicine Reports. 2018;17(5):6681-6690. doi:10.3892/mmr.2018.8657	cell culture		functional assay	
China	Ying X, Chen X, Feng Y, Xu HZ, Chen H, Yu K, Cheng S, Peng L. Myricetin enhances osteogenic differentiation through the activation of canonical Wnt/β-catenin signaling in human bone marrow stromal cells. Eur J Pharmacol. 2014 Sep 5;738:22-30. doi: 10.1016/j.ejphar.2014.04.049. Epub 2014 May 27. PMID: 24876056.	cell culture		administration assay	
China	Ying X, Chen X, Liu H, Nie P, Shui X, Shen Y, Yu K, Cheng S. Silibinin alleviates high glucose-suppressed osteogenic differentiation of human bone marrow stromal cells via antioxidant effect and PI3K/Akt signaling. Eur J Pharmacol. 2015 Oct 15;765:394-401. doi: 10.1016/j.ejphar.2015.09.005. Epub 2015 Sep 8. PMID: 26362750.	cell culture		functional assay; administration assay	
China	Zhang L, Li X, Kong X, Jin H, Han Y, Xie Y. Effects of the NF-κB/p53 signaling pathway on intervertebral disc nucleus pulposus degeneration. Mol Med Rep. 2020 Sep;22(3):1821-1830. doi: 10.3892/mmr.2020.11288. Epub 2020 Jun 30. PMID: 32705171; PMCID: PMC7411364.	cell culture		functional assay	
China	Zhang, F., et al. "PARK7 Promotes Repair in Early Steroid-Induced Osteonecrosis of the Femoral Head by Enhancing Resistance to Stress-Induced Apoptosis in Bone Marrow Mesenchymal Stem Cells Via Regulation of the Nrf2 Signaling Pathway." Cell Death and Disease, vol. 12, no. 10, 2021. SCOPUS, www.scopus.com, doi:10.1038/s41419-021-04226-1.	animals		functional assay	
China	Zhang, J., et al. "Protective Effects of 2,3,5,4-Tetrahydroxystilbene-2-o-β-D-Glucoside Against Osteoporosis: Current Knowledge and Proposed Mechanisms." International Journal of Rheumatic Diseases, vol. 21, no. 8, 2018, pp. 1504-1513. SCOPUS, www.scopus.com, doi:10.1111/1756-185X.13357.	animals		administration assay	
China	Zhang, Shuai; Guo, Weiwei; Zhao, Xin; Li, Peng. Downregulation of Prdx2 Protects Osteoporosis Rats by Regulating Receptor Activator of Nuclear Factor Kappa-B/Osteoprotegerin Pathway. Journal of Biomaterials and Tissue Engineering, Volume 9, Number 6, June 2019, pp. 839-844(6). https://doi.org/10.1166/jbt.2019.2042	animals		functional assay	
China	Zhao J, Zhang M, Quan Z, Deng L, Li Y, He B. Systematic Influence of Circulating Bilirubin Levels on Osteoporosis. Front Endocrinol (Lausanne). 2021 Aug 26;12:719920. doi: 10.3389/fendo.2021.719920. PMID: 34539572; PMCID: PMC8447935.			Mendelian randomization analysis	
China	Zhao T, Chen J, Liu S, et al. Transcriptome analysis of Fusobacterium nucleatum reveals differential gene expression patterns in the biofilm versus planktonic cells. Biochemical & Biophysical Research Communications. 2022;593:151-157. doi:10.1016/j.bbrc.2021.11.075	microbiota	periodontitis	transcriptomics	
China	Zhou J, Wang F, Ma Y, Wei F. Vitamin D3 contributes to enhanced osteogenic differentiation of MSCs under oxidative stress condition via activating the endogenous antioxidant system. Osteoporosis International. 2018;29(8):1917-1926. doi:10.1007/s00198-018-4547-0	cell culture		administration assay	
China	Zhou L, Wu T. A Network Pharmacology-Based Study on Vital Pharmacological Pathways and Targets of Eucommiae Cortex Acting on Osteoporosis. BioMed Research International. March 2022:1-14. doi:10.1155/2022/8510842	cell culture			review
China	Zhou N, Lin X, Dong W, Huang W, Jiang W, Lin L, Qiu Q, Zhang X, Shen J, Song Z, Liang X, Hao J, Wang D, Hu Z. SIRT1 alleviates senescence of degenerative human intervertebral disc cartilage endo-plate cells via the p53/p21 pathway. Sci Rep. 2016 Mar 4;6:22628. doi: 10.1038/srep22628. PMID: 26940203; PMCID: PMC4778056.	cell culture		functional assay	

China	Zhou Z, Lu Y, Wang Y, Du L, Zhang Y, Tao J. Let-7c regulates proliferation and osteodifferentiation of human adipose-derived mesenchymal stem cells under oxidative stress by targeting SCD-1. Am J Physiol Cell Physiol. 2019 Jan 1;316(1):C57-C69. doi: 10.1152/ajpcell.00211.2018. Epub 2018 Oct 31. PMID: 30379578.	cell culture	functional assay		
China	Zhu S, Wei W, Liu Z, Yang Y, Jia H. Tanshinone-IIA attenuates the deleterious effects of oxidative stress in osteoporosis through the NF-κB signaling pathway. Molecular Medicine Reports. 2018;17(5):6969-6976. doi:10.3892/mmr.2018.8741	cell culture	administration assay		
China	Zhu W, Xie K, Yang J, Li L, Wang X, Xu L, Fang S. Diagnosis of Klippel-Trenaunay syndrome and extensive heterotopic ossification in a patient with a femoral fracture: a case report and literature review. BMC Musculoskelet Disord. 2020 Apr 11;21(1):223. doi: 10.1186/s12891-020-03224-2. PMID: 32278353; PMCID: PMC7149888.				case report; review
China	Zou, D. -., et al. "TRIM33 Protects Osteoblasts from Oxidative Stress-Induced Apoptosis in Osteoporosis by Inhibiting FOXO3a Ubiquitylation and Degradation." Aging Cell, vol. 20, no. 7, 2021. SCOPUS, www.scopus.com, doi:10.1111/acel.13367.	animals	functional assay		
China	Zuo R, Liu M, Wang Y, et al. BM-MSC-derived exosomes alleviate radiation-induced bone loss by restoring the function of recipient BM-MSCs and activating Wnt/β-catenin signaling. Stem Cell Research & Therapy. 2019;10(1):N.PAG. doi:10.1186/s13287-018-1121-9	animals	functional assay		
China	周年, 刘波, 徐彭. 氧化应激与骨质疏松症的研究进展. Chinese Journal of Osteoporosis / Zhongguo Guzhi Shusong Zazhi. 2014;20(12):1485-1489. doi:10.3969/j.issn.1006-7108.2014.12.025			chinese	review
China	戴梦竹, 任路, 何信用, 王群, 徐宁阳, 陈文娜. 基于网络药理学的三仙汤治疗骨质疏松症作用机制研究. Chinese Journal of Osteoporosis / Zhongguo Guzhi Shusong Zazhi. 2021;27(6):831-837. doi:10.3969/j.issn.1006-7108.2021.06.010		pharmacology	chinese	database research
China	李超, 赵剑波, 陈俊推, 耿玲, 何宁, 赵浩东. 金天格胶囊对h2o2诱导的小鼠成骨细胞mc3t3-E1 氧化应激损伤及炎症因子的作用. Chinese Journal of Osteoporosis / Zhongguo Guzhi Shusong Zazhi. 2022;28(10):1448-1532. doi:10.3969/j.issn.1006-7108.2022.10.008	cell culture	administration assay	chinese	
China	程韶, 舒冰, 赵永见, et al. 氧化应激对骨重建的影响. Chinese Journal of Osteoporosis / Zhongguo Guzhi Shusong Zazhi. 2019;25(10):1478-1482. doi:10.3969/j.issn.1006-7108.2019.10.024			chinese	review
China	罗 臻, 黄禹僖, 柴生颋, 李飞龙, 陈群群. 补肾健脾活血方与靶点密切相关组蛋白去甲基化酶jmjd2b 在骨质疏松症中 促成骨分化：体外细胞实验验证. Chinese Journal of Tissue Engineering Research / Zhongguo zu zhi gong cheng yan jiu. 2022;26(29):4643-4650. doi:10.12307/2022.904	cell culture	administration assay		
China	贾珍, 顾抚顺, 王爱国. 睡眠与骨质疏松关系的研究进展. Chinese Journal of Osteoporosis / Zhongguo Guzhi Shusong Zazhi. 2021;27(3):463-468. doi:10.3969/j.issn.1006-7108.2021.03.031			chinese	review
China	闫立言, 韩萧男, 寇红伟, et al. 褪黑素防治骨质疏松症的作用与应用现状. Chinese Journal of Tissue Engineering Research / Zhongguo zu zhi gong cheng yan jiu. 2023;27(14):2222-2228. doi:10.12307/2023.430			chinese	review

China	顾 超, 陈维凯, 刘 滔, 杨惠林, 何 帆. 骨髓间充质干细胞线粒体损伤影响其成骨分化的潜能. Chinese Journal of Tissue Engineering Research / Zhongguo zu zhi gong cheng yan jiu. 2022;26(31):4921-4927. doi:10.12307/2022.727	cell culture		mitochondria	
Czech Republic	Krizkova S, Kepinska M, Emri G, et al. Microarray analysis of metallothioneins in human diseases—A review. Journal of Pharmaceutical & Biomedical Analysis. 2016;117:464-473. doi:10.1016/j.jpba.2015.09.031		cancer	transcriptomics	review
Denmark	Ali D, Chen L, Kowal JM, Okla M, Manikandan M, AlShehri M, AlMana Y, AlObaidan R, AlOtaibi N, Hamam R, Alajez NM, Aldahmash A, Kassem M, Alfayez M. Resveratrol inhibits adipocyte differentiation and cellular senescence of human bone marrow stromal stem cells. Bone. 2020 Apr;133:115252. doi: 10.1016/j.bone.2020.115252. Epub 2020 Jan 21. PMID: 31978617.	cell culture		administration assay	
Egypt	Alkhedaide, A., et al. "Chronic Effects of Soft Drink Consumption on the Health State of Wistar Rats: A Biochemical, Genetic and Histopathological Study." Molecular Medicine Reports, vol. 13, no. 6, 2016, pp. 5109-5117. SCOPUS, www.scopus.com, doi:10.3892/mmr.2016.5199.	animals		chronic soft drink consumption (SDC	
Egypt	Ameen O, Yassien RI, Naguib YM. Activation of FoxO1/SIRT1/RANKL/OPG pathway may underlie the therapeutic effects of resveratrol on aging-dependent male osteoporosis. BMC Musculoskeletal Disorders. 2020;21(1):1-14. doi:10.1186/s12891-020-03389-w	animals		functional assay	
Egypt	Clayton, Z. S., et al. "Chronic Ethanol Consumption does Not Reduce True Bone Density in Male Wistar Rats." Alcohol, vol. 93, 2021, pp. 17-23. SCOPUS, www.scopus.com, doi:10.1016/j.alcohol.2021.02.003.	animals		ethanol	
Egypt	Elghareeb MM, Elshopakey GE, Elkhooly TA, Salama B, Samy A, Bazer FW, Elmetwally MA, Almutairi MH, Aleya L, Abdel-Daim MM, Rezk S. Estradiol and zinc-doped nano hydroxyapatite as therapeutic agents in the prevention of osteoporosis; oxidative stress status, inflammation, bone turnover, bone mineral density, and histological alterations in ovariectomized rats. Front Physiol. 2022 Sep 19;13:989487. doi: 10.3389/fphys.2022.989487. PMID: 36200054; PMCID: PMC9527315.	animals		administration assay	
Egypt	El-Makawy AI, Ibrahim FM, Mabrouk DM, Abdel-Aziem SH, Sharaf HA, Ramadan MF. Efficiency of turnip bioactive lipids in treating osteoporosis through activation of Osterix and suppression of Cathepsin K and TNF-α signaling in rats. Environ Sci Pollut Res Int. 2020 Jun;27(17):20950-20961. doi: 10.1007/s11356-020-08540-7. Epub 2020 Apr 6. PMID: 32253695.	animals		functional assay	
Egypt	Erfan, O. S., Salem, Y. G., El-Shahat, M. A., Awadin, W. F., Eltahry, H. & Eldesoqui, M. (2022). Potential benefits of dihydroartemisinin in suppression of dexamethasone induced osteoporosis, osteoclast formation and RANKL induced signaling pathways in adult female albino rat. European Journal of Anatomy, 26(5), 509-521. https://doi.org/10.52083/ituy9072	animals		administration assay	
Egypt	Ezzat, S., et al. "Autophagy in Osteoporosis: Relation to Oxidative Stress." Journal of Cellular Biochemistry, vol. 120, no. 2, 2019, pp. 2560-2568. SCOPUS, www.scopus.com, doi:10.1002/jcb.27552.			functional assay	
Egypt	GamalEl Din SF, Rashed LA, Alghobary HA, Tawfik LT, ElSheemy MS. Are the Cavernous Tissue and Serum Levels of Micro RNAs 200a and 206 Elevated in Patients With Refractory			transcriptomics	

	Veno-occlusive Erectile Dysfunction? A Comparative Study. Urology. 2017 Oct;108:108-113. doi: 10.1016/j.urology.2017.07.020. Epub 2017 Jul 26. PMID: 28755962.				
Egypt	Hamed, E. M., et al. "Recent Progress in Gene Therapy and Other Targeted Therapeutic Approaches for Beta Thalassemia." Current Drug Targets, vol. 20, no. 16, 2019, pp. 1603-1623. SCOPUS, www.scopus.com, doi:10.2174/1389450120666190726155733.				review
Egypt	Ragab, S. M., E. A. Badr, and A. S. Ibrahim. "Evaluation of Glutathione-S-Transferase P1 Polymorphism and its Relation to Bone Mineral Density in Egyptian Children and Adolescents with Beta-Thalassemia Major." Mediterranean Journal of Hematology and Infectious Diseases, vol. 8, no. 1, 2016. SCOPUS, www.scopus.com, doi:10.4084/mjhid.2016.004.	children			
EUA	Dalbeth N, Topless R, Flynn T, Cadzow M, Bolland MJ, Merriman TR. Mendelian randomization analysis to examine for a causal effect of urate on bone mineral density. J Bone Miner Res. 2015 Jun;30(6):985-91. doi: 10.1002/jbmr.2434. PMID: 25502344.			Mendelian randomization analysis	
EUR - EUA	Justice AE, Winkler TW, Feitosa MF, Graff M, Fisher VA, Young K, Barata L, Deng X, Czajkowski J, Hadley D, Ngwa JS, Ahluwalia TS, Chu AY, Heard-Costa NL, Lim E, Perez J, Eicher JD, Kutalik Z, Xue L, Mahajan A, Renström F, Wu J, Qi Q, Ahmad S, Alfred T, Amin N, Bielak LF, Bonnefond A, Bragg J, Cadby G, Chittani M, Coggeshall S, Corre T, Direk N, Eriksson J, Fischer K, Gorski M, Neergaard Harder M, Horikoshi M, Huang T, Huffman JE, Jackson AU, Justesen JM, Kanoni S, Kinnunen L, Kleber ME, Komulainen P, Kumari M, Lim U, Luan J, Lyytikäinen LP, Mangino M, Manichaikul A, Marten J, Middelberg RPS, Müller-Nurasyid M, Navarro P, Pérusse L, Pervjakova N, Sarti C, Smith AV, Smith JA, Stančáková A, Strawbridge RJ, Stringham HM, Sung YJ, Tanaka T, Teumer A, Trompet S, van der Laan SW, van der Most PJ, Van Vliet-Ostaptchouk JV, Vedantam SL, Verweij N, Vink JM, Vitart V, Wu Y, Yengo L, Zhang W, Hua Zhao J, Zimmermann ME, Zubair N, Abecasis GR, Adair LS, Afaq S, Afzal U, Bakker SJL, Bartz TM, Beilby J, Bergman RN, Bergmann S, Biffar R, Blangero J, Boerwinkle E, Bonnycastle LL, Bottinger E, Braga D, Buckley BM, Buyske S, Campbell H, Chambers JC, Collins FS, Curran JE, de Borst GJ, de Craen AJM, de Geus EJC, Dedoussis G, Delgado GE, den Ruijter HM, Eiriksdottir G, Eriksson AL, Esko T, Faul JD, Ford I, Forrester T, Gertow K, Gigante B, Glorioso N, Gong J, Grallert H, Grammer TB, Grarup N, Haitjema S, Hallmans G, Hamsten A, Hansen T, Harris TB, Hartman CA, Hassinen M, Hastie ND, Heath AC, Hernandez D, Hindorff L, Hocking LJ, Hollensted M, Holmen OL, Homuth G, Jan Hottenga J, Huang J, Hung J, Hutri-Kähönen N, Ingelsson E, James AL, Jansson JO, Jarvelin MR, Jhun MA, Jørgensen ME, Juonala M, Kähönen M, Karlsson M, Koistinen HA, Kolcic I, Kolovou G, Kooperberg C, Krämer BK, Kuusisto J, Kvaløy K, Lakka TA, Langenberg C, Launer LJ, Leander K, Lee NR, Lind L, Lindgren CM, Linneberg A, Lobbens S, Loh M, Lorentzon M, Luben R, Lubke G, Ludolph-Donislawski A, Lupoli S, Madden PAF, Männikkö R, Marques-Vidal P, Martin NG, McKenzie CA, McKnight B, Mellström D, Menni C, Montgomery GW, Musk AB, Narisu N, Nauck M, Nolte IM, Oldehinkel AJ, Olden M, Ong KK, Padmanabhan S, Peyser PA, Pisinger C, Porteous DJ, Raitakari OT, Rankinen T, Rao DC, Rasmussen-Torvik LJ, Rawal R, Rice T, Ridker PM, Rose LM, Bien SA, Rudan I, Sanna S, Sarzynski MA, Sattar N, Savonen K, Schlessinger D, Scholtens S, Schurmann C, Scott RA, Sennblad B, Siemelink MA, Silbernagel G, Slagboom PE, Snieder H, Staessen JA, Stott DJ, Swertz MA, Swift AJ, Taylor KD, Tayo BO, Thorand B, Thuillier D, Tuomilehto J, Uitterlinden		obesity		

	AG, Vandenput L, Vohl MC, Völzke H, Vonk JM, Waeber G, Waldenberger M, Westendorp RGJ, Wild S, Willemsen G, Wolffenbuttel BHR, Wong A, Wright AF, Zhao W, Zillikens MC, Baldassarre D, Balkau B, Bandinelli S, Böger CA, Boomsma DI, Bouchard C, Bruinenberg M, Chasman DI, Chen YD, Chines PS, Cooper RS, Cucca F, Cusi D, Faire U, Ferrucci L, Franks PW, Froguel P, Gordon-Larsen P, Grabe HJ, Gudnason V, Haiman CA, Hayward C, Hveem K, Johnson AD, Wouter Jukema J, Kardia SLR, Kivimaki M, Kooner JS, Kuh D, Laakso M, Lehtimäki T, Marchand LL, März W, McCarthy MI, Metspalu A, Morris AP, Ohlsson C, Palmer LJ, Pasterkamp G, Pedersen O, Peters A, Peters U, Polasek O, Psaty BM, Qi L, Rauramaa R, Smith BH, Sørensen TIA, Strauch K, Tiemeier H, Tremoli E, van der Harst P, Vestergaard H, Vollenweider P, Wareham NJ, Weir DR, Whitfield JB, Wilson JF, Tyrrell J, Frayling TM, Barroso I, Boehnke M, Deloukas P, Fox CS, Hirschhorn JN, Hunter DJ, Spector TD, Strachan DP, van Duijn CM, Heid IM, Mohlke KL, Marchini J, Loos RJF, Kilpeläinen TO, Liu CT, Borecki IB, North KE, Cupples LA. Genome-wide meta-analysis of 241,258 adults accounting for smoking behaviour identifies novel loci for obesity traits. Nat Commun. 2017 Apr 26;8:14977. doi: 10.1038/ncomms14977. PMID: 28443625; PMCID: PMC5414044.			
Finland	Salminen A. Aryl hydrocarbon receptor (AhR) reveals evidence of antagonistic pleiotropy in the regulation of the aging process. Cellular & Molecular Life Sciences. 2022;79(9):1-21. doi:10.1007/s00018-022-04520-x	aging		review
France	Collin P, Lomri A, Marie PJ. Expression and activity of NAD(P)H:quinone oxidoreductase (NMO1) in human osteoblastic cells. Bone. 2001 Jan;28(1):9-13. doi: 10.1016/s8756-3282(00)00435-x. PMID: 11165937.	cell culture	transcriptomics	
France	Lahaye, C., et al. "Does Iron Overload in Metabolic Syndrome Affect Macrophage Profile? A Case Control Study." Journal of Trace Elements in Medicine and Biology, vol. 67, 2021. SCOPUS, www.scopus.com, doi:10.1016/j.jtemb.2021.126786.	cell culture	administration assay	
France	Tagliaferri C, Davicco M-J, Lebecque P, et al. Olive Oil and Vitamin D Synergistically Prevent Bone Loss in Mice. PLoS ONE. 2014;9(12):1-19. doi:10.1371/journal.pone.0115817	animals	administration assay	
Germany	Habig C, Geffers R, Distl O. A Replication Study for Genome-Wide Gene Expression Levels in Two Layer Lines Elucidates Differentially Expressed Genes of Pathways Involved in Bone Remodeling and Immune Responsiveness. PLoS ONE. 2014;9(6):1-8. doi:10.1371/journal.pone.0098350	cell culture	transcriptomics	
Germany	He Y, Wuertz-Kozak K, Kuehl LK, Wippert P-M. Extracellular Vesicles: Potential Mediators of Psychosocial Stress Contribution to Osteoporosis? International Journal of Molecular Sciences. 2021;22(11):5846. doi:10.3390/ijms22115846		vesicles	review
Germany	Hofbauer LC, Brueck CC, Shanahan CM, Schoppet M, Dobnig H. Vascular calcification and osteoporosis—from clinical observation towards molecular understanding. Osteoporosis International. 2007;18(3):251-259. doi:10.1007/s00198-006-0282-z			review
Germany	Lackner, I., et al. "Midkine is Elevated After Multiple Trauma and Acts Directly on Human Cardiomyocytes by Altering their Functionality and Metabolism." Frontiers in Immunology, vol. 10, no. AUG, 2019. SCOPUS, www.scopus.com, doi:10.3389/fimmu.2019.01920.	cell culture	functional assay	
Germany	Leitzbach, D., et al. "Restoration of Endothelial Function Via Enhanced Nitric Oxide Synthesis After Long-Term Treatment of Raloxifene in Adult Hypertensive Rats." Arzneimittel-	animals	functional assay	

	Forschung/Drug Research, vol. 55, no. 2, 2005, pp. 86-92. SCOPUS, www.scopus.com, doi:10.1055/s-0031-1296828.					
Germany	Mentlein, R. "Targeting Pleiotropin to Treat Osteoarthritis." Expert Opinion on Therapeutic Targets, vol. 11, no. 7, 2007, pp. 861-867. SCOPUS, www.scopus.com, doi:10.1517/14728222.11.7.861.			functional assay		review
Germany	Schreckenberg R, Wenzel S, da Costa Rebelo RM, Röthig A, Meyer R, Schlüter KD. Cell-specific effects of nitric oxide deficiency on parathyroid hormone-related peptide (PTHrP) responsiveness and PTH1 receptor expression in cardiovascular cells. Endocrinology. 2009 Aug;150(8):3735-41. doi: 10.1210/en.2008-1585. Epub 2009 Apr 2. PMID: 19342458.	animals	cardiac risk	functional assay		
Germany	Tohidnezhad M, Wruck CJ, Slowik A, Kweider N, Beckmann R, Bayer A, Houben A, Brandenburg LO, Varoga D, Sönmez TT, Stoffel M, Jahr H, Lippross S, Pufe T. Role of platelet-released growth factors in detoxification of reactive oxygen species in osteoblasts. Bone. 2014 Aug;65:9-17. doi: 10.1016/j.bone.2014.04.029. Epub 2014 May 4. PMID: 24798492.	cell culture		functional assay		
Germany	Wuertz-Kozak, K., et al. "Effects of Early Life Stress on Bone Homeostasis in Mice and Humans." International Journal of Molecular Sciences, vol. 21, no. 18, 2020, pp. 1-25. SCOPUS, www.scopus.com, doi:10.3390/ijms21186634.	animals		functional assay		
Hong Kong	Liu B, Ghosh S, Yang X, Zheng H, Liu X, Wang Z, Jin G, Zheng B, Kennedy BK, Suh Y, Kaeberlein M, Tryggvason K, Zhou Z. Resveratrol rescues SIRT1-dependent adult stem cell decline and alleviates progeroid features in laminopathy-based progeria. Cell Metab. 2012 Dec 5;16(6):738-50. doi: 10.1016/j.cmet.2012.11.007. PMID: 23217256.	cell culture		administration assay		
Hungary	Nath A, Molnár MA, Csighy A, Kőszegi K, Galambos I, Huszár KP, Koris A, Vatai G. Biological Activities of Lactose-Based Prebiotics and Symbiosis with Probiotics on Controlling Osteoporosis, Blood-Lipid and Glucose Levels. Medicina (Kaunas). 2018 Dec 3;54(6):98. doi: 10.3390/medicina54060098. PMID: 30513975; PMCID: PMC6306850.					review
India	Agrawal N, Verma K, Baghel D, Chauhan A, Prasad DN, Sharma SK, Kohli E. Effects of extremely low-frequency electromagnetic field on different developmental stages of Drosophila melanogaster. Int J Radiat Biol. 2021;97(11):1606-1616. doi: 10.1080/09553002.2021.1969465. Epub 2021 Aug 31. PMID: 34402374.	animals		functional assay		
India	Choudhary, D., et al. "Prevention of Articular Cartilage Degeneration in a Rat Model of Monosodium Iodoacetate Induced Osteoarthritis by Oral Treatment with Withaferin A." Biomedicine and Pharmacotherapy, vol. 99, 2018, pp. 151-161. SCOPUS, www.scopus.com, doi:10.1016/j.biopha.2017.12.113.	animals		administration assay		
India	Deka, R. S., et al. "Chromium Supplements in the Feed for Lactating Murrah Buffaloes (Bubalus Bubalis): Influence on Nutrient Utilization, Lactation Performance, and Metabolic Responses." Biological Trace Element Research, vol. 168, no. 2, 2015, pp. 362-371. SCOPUS, www.scopus.com, doi:10.1007/s12011-015-0372-x.	animals		diet intervention		
India	Dixit M, Singh K, Prakash R, Singh D. Functional block of IL-17 cytokine promotes bone healing by augmenting FOXO1 and ATF4 activity in cortical bone defect model. Osteoporosis International. 2017;28(7):2207-2220. doi:10.1007/s00198-017-4012-5	animals		functional assay		
India	Khan NM, Sandur SK, Checker R, Sharma D, Poduval TB, Sainis KB. Pro-oxidants ameliorate radiation-induced apoptosis through activation of the calcium–ERK1/2–Nrf2 pathway. Free Radical Biology & Medicine. 2011;51(1):115-128. doi:10.1016/j.freeradbiomed.2011.03.037	cell culture		administration assay		

India	Kour, H., et al. "Evaluation of the Wound Healing Activity of Ethanolic Extract of Bergenia Ciliata (Haw.) Sternb. Rhizome with Excision Wound Model in Wistar Rats." Journal of Ethnopharmacology, vol. 281, 2021. SCOPUS, www.scopus.com, doi:10.1016/j.jep.2021.114527.	animals		administration assay	
India	Mittal M, Bhagwati S, Siddiqi MI, Chattopadhyay N. A critical assessment of the potential of pharmacological modulation of aldehyde dehydrogenases to treat the diseases of bone loss. European Journal of Pharmacology. 2020;886:N.PAG. doi:10.1016/j.ejphar.2020.173541	animals			review
India	Mittal M, Pal S, China SP, Porwal K, Dev K, Shrivastava R, Raju KS, Rashid M, Trivedi AK, Sanyal S, Wahajuddin M, Bhaduria S, Maurya R, Chattopadhyay N. Pharmacological activation of aldehyde dehydrogenase 2 promotes osteoblast differentiation via bone morphogenetic protein-2 and induces bone anabolic effect. Toxicol Appl Pharmacol. 2017 Feb 1;316:63-73. doi: 10.1016/j.taap.2016.12.013. Epub 2016 Dec 23. PMID: 28017615.	animals		administration assay	
India	Mittal SPK, Khole S, Jagadish N, et al. Andrographolide protects liver cells from H2O2 induced cell death by upregulation of Nrf-2/HO-1 mediated via adenosine A2a receptor signalling. BBA - General Subjects. 2016;1860(11a):2377-2390. doi:10.1016/j.bbagen.2016.07.005	cell cuture		functional assay	
India	Nilawar, S., and K. Chatterjee. "Surface Decoration of Redox-Modulating Nanoceria on 3D-Printed Tissue Scaffolds Promotes Stem Cell Osteogenesis and Attenuates Bacterial Colonization." Biomacromolecules, vol. 23, no. 1, 2022, pp. 226-239. SCOPUS, www.scopus.com, doi:10.1021/acs.biomac.1c01235.			3D printing therapy	
India	Pal S, Porwal K, Khanna K, Gautam MK, Malik MY, Rashid M, Macleod RJ, Wahajuddin M, Parameswaran V, Bellare JR, Chattopadhyay N. Oral dosing of pentoxifylline, a pan-phosphodiesterase inhibitor restores bone mass and quality in osteopenic rabbits by an osteogenic mechanism: A comparative study with human parathyroid hormone. Bone. 2019 Jun;123:28-38. doi: 10.1016/j.bone.2019.03.010. Epub 2019 Mar 9. PMID: 30858147.	animals		functional assay	
India	Pandiarajan S, Samuel S, Loganathan T, et al. Pila globosa snail extract inhibits osteoclast differentiation via downregulation of nuclear factor κB and nuclear factor of activated T-Cells c1 signaling pathways. Pharmacognosy Magazine. 2019;15(64):298-306. doi:10.4103/pm.pm_39_19	animals		administration assay	
India	Sharan K, Mishra JS, Swarnkar G, Siddiqui JA, Khan K, Kumari R, Rawat P, Maurya R, Sanyal S, Chattopadhyay N. A novel quercetin analogue from a medicinal plant promotes peak bone mass achievement and bone healing after injury and exerts an anabolic effect on osteoporotic bone: the role of aryl hydrocarbon receptor as a mediator of osteogenic action. J Bone Miner Res. 2011 Sep;26(9):2096-111. doi: 10.1002/jbmr.434. PMID: 21638315.	animals		administration assay	
India	Thummuri D, Naidu V, Chaudhari P. Carnosic acid attenuates RANKL-induced oxidative stress and osteoclastogenesis via induction of Nrf2 and suppression of NF-κB and MAPK signalling. Journal of Molecular Medicine. 2017;95(10):1065-1076. doi:10.1007/s00109-017-1553-1	animals		administration assay	
Indonesia	Hendrianingtyas, M., B. Rachmawati, and P. Adhipireno. "The Differences of Parathyroid Hormone, Vitamin D, and Calcium Ion between Patients with Controlled and Uncontrolled Diabetes Mellitus." Pakistan Journal of Medical and Health Sciences, vol. 14, no. 4, 2021, pp. 1794-1797. SCOPUS, www.scopus.com.		T2D	serum levels	

Iran	Abedpoor N, Taghian F, Hajibabaie F. Physical activity ameliorates the function of organs via adipose tissue in metabolic diseases. Acta Histochemica. 2022;124(2):N.PAG. doi:10.1016/j.acthis.2022.151844		exercise		review
Iran	Gholamrezayi, A., et al. "The Effect of Cornus Mas Fruit Extract Consumption on Lipid Profile, Glycemic Indices, and Leptin in Postmenopausal women— A Randomized Clinical Trial." Phytotherapy Research, vol. 33, no. 11, 2019, pp. 2979-2988. SCOPUS, www.scopus.com, doi:10.1002/ptr.6476.		menopause	administration assay	
Iran.	Emamgholipour S, Hossein-Nezhad A, Sahraian MA, Askarisadr F, Ansari M. Evidence for possible role of melatonin in reducing oxidative stress in multiple sclerosis through its effect on SIRT1 and antioxidant enzymes. Life Sci. 2016 Jan 15;145:34-41. doi: 10.1016/j.lfs.2015.12.014. Epub 2015 Dec 8. PMID: 26679105.	cell culture		administration assay	
Iraq	Hassan, M. A. A., and N. M. H. AL-Ghaban. "Immunohistochemical Localization of Bone Morphogenic Protein-2 in Extracted Tooth Socket Treated by Local Application of Grape Seeds Oil in Rabbits." Biochemical and Cellular Archives, vol. 20, no. 1, 2020, pp. 581-589. SCOPUS, www.scopus.com, doi:10.35124/bca.2020.20.1.581.	animals		administration assay	
Israel	Mandel S, Packer L, Youdim MBH, Weinreb O. Proceedings from the “Third International Conference on Mechanism of Action of Nutraceuticals.” Journal of Nutritional Biochemistry. 2005;16(9):513-520. doi:10.1016/j.jnutbio.2005.03.001			diet supplements	review
Israel	Mijiritsky E, Ferroni L, Gardin C, et al. Presence of ROS in Inflammatory Environment of Peri-Implantitis Tissue: In Vitro and In Vivo Human Evidence. Journal of Clinical Medicine. 2020;9(1):38. doi:10.3390/jcm9010038	cell culture	inflammation		
Israel	Savion N, Abu-Kheit R, Kotev-Emeth S, Levine A, Broday L, Gabet Y. 239 - S-Allylmercapto-N-Acetylcysteine Protects Caenorhabditis Elegans and Cultured Stromal Bone Marrow Cells from Oxidative Stress and Improves Bone Microarchitecture of Healthy and Diabetic Mice. Free Radical Biology & Medicine. 2016;100:S109. doi:10.1016/j.freeradbiomed.2016.10.280	animals		functional assay	
Italy	Boccanegra, B., et al. "Safety Issues and Harmful Pharmacological Interactions of Nutritional Supplements in Duchenne Muscular Dystrophy: Considerations for Standard of Care and Emerging Virus Outbreaks." Pharmacological Research, vol. 158, 2020. SCOPUS, www.scopus.com, doi:10.1016/j.phrs.2020.104917.				review
Italy	Brandi ML, Hukkanen M, Umeda T, Moradi-Bidhendi N, Bianchi S, Gross SS, Polak JM, MacIntyre I. Bidirectional regulation of osteoclast function by nitric oxide synthase isoforms. Proc Natl Acad Sci U S A. 1995 Mar 28;92(7):2954-8. doi: 10.1073/pnas.92.7.2954. PMID: 7535933; PMCID: PMC42337.	animals		functional assay	
Italy	Calò L, Giannini S, Bonvicini P, Nobile M, Cantaro S, Plebani M, Semplicini A, D'Angelo A, Crepaldi G. Idiopathic hypercalciuria: O2(-)NO relationship and altered bone metabolism. J Endocrinol Invest. 2000 Feb;23(2):78-83. doi: 10.1007/BF03343683. PMID: 10800759.	cell culture		functional assay	
Italy	Carnovali M, Luzi L, Terruzzi I, Banfi G, Mariotti M. Liquiritigenin Reduces Blood Glucose Level and Bone Adverse Effects in Hyperglycemic Adult Zebrafish. Nutrients. 2019 May 9;11(5):1042. doi: 10.3390/nu11051042. PMID: 31075971; PMCID: PMC6566992.	animals		administration assay	
Italy	Di Bari F, Catalano A, Bellone F, Martino G, Benvenga S. Vitamin D, Bone Metabolism, and Fracture Risk in Polycystic Ovary Syndrome. Metabolites. 2021 Feb 18;11(2):116. doi: 10.3390/metabo11020116. PMID: 33670644; PMCID: PMC7922814.				review

Italy	Gennari L, Merlotti D, Figura N, Mingiano C, Franci MB, Lucani B, Picchioni T, Alessandri M, Campagna MS, Gonnelli S, Bianciardi S, Materozzi M, Caffarelli C, Gonnelli S, Nuti R. Infection by CagA-Positive Helicobacter pylori Strains and Bone Fragility: A Prospective Cohort Study. J Bone Miner Res. 2021 Jan;36(1):80-89. doi: 10.1002/jbmr.4162. Epub 2020 Aug 31. PMID: 32790186.		Helicobacter pylori		
Italy	Menghini, L., et al. "A Natural Formula Containing Lactoferrin, Equisetum Arvensis, Soy Isoflavones and Vitamin D3 Modulates Bone Remodeling and Inflammatory Markers in Young and Aged Rats." Journal of Biological Regulators and Homeostatic Agents, vol. 30, no. 4, 2016, pp. 985-996. SCOPUS, www.scopus.com.	animals		administration assay	
Italy	Pagano G, Talamanca AA, Castello G, d'Ischia M, Pallardó FV, Petrović S, Porto B, Tiano L, Zatterale A. Bone marrow cell transcripts from Fanconi anaemia patients reveal in vivo alterations in mitochondrial, redox and DNA repair pathways. Eur J Haematol. 2013 Aug;91(2):141-51. doi: 10.1111/ejh.12131. Epub 2013 Jun 15. PMID: 23646927.		anaemia	transcriptomics	
Italy	Rendina D, De Filippo G, Iannuzzo G, Abate V, Strazzullo P, Falchetti A. Idiopathic Osteoporosis and Nephrolithiasis: Two Sides of the Same Coin? Int J Mol Sci. 2020 Oct 31;21(21):8183. doi: 10.3390/ijms21218183. PMID: 33142950; PMCID: PMC7662860.				review
Italy	Renzo, L. D., et al. "A Hazelnut-Enriched Diet Modulates Oxidative Stress and Inflammation Gene Expression without Weight Gain." Oxidative Medicine and Cellular Longevity, vol. 2019, 2019. SCOPUS, www.scopus.com, doi:10.1155/2019/4683723.			diet intervention	
Italy	Rondanelli, M., et al. "Focus on Pivotal Role of Dietary Intake (Diet and Supplement) and Blood Levels of Tocopherols and Tocotrienols in Obtaining Successful Aging." International Journal of Molecular Sciences, vol. 16, no. 10, 2015, pp. 23227-23249. SCOPUS, www.scopus.com, doi:10.3390/ijms161023227.			diet supplements	review
Italy	Terruzzi I, Montesano A, Senesi P, Villa I, Ferraretto A, Bottani M, Vacante F, Spinello A, Bolamperti S, Luzi L, Rubinacci A. L-Carnitine Reduces Oxidative Stress and Promotes Cells Differentiation and Bone Matrix Proteins Expression in Human Osteoblast-Like Cells. Biomed Res Int. 2019 Jan 20;2019:5678548. doi: 10.1155/2019/5678548. PMID: 30800672; PMCID: PMC6360619.	cell culture		administration assay	
Italy	Tinti L, Niccolini S, Lamboglia A, Pascarelli NA, Cervone R, Fioravanti A. Raloxifene protects cultured human chondrocytes from IL-1β induced damage: a biochemical and morphological study. Eur J Pharmacol. 2011 Nov 16;670(1):67-73. doi: 10.1016/j.ejphar.2011.08.027. Epub 2011 Sep 5. PMID: 21920358.	cell culture		administration assay	
Italy	Venditti P, Stefano L, Meo S. Vitamin E management of oxidative damage-linked dysfunctions of hyperthyroid tissues. Cellular & Molecular Life Sciences. 2013;70(17):3125-3144. doi:10.1007/s00018-012-1217-9				review
Italy	Versari S, Longinotti G, Barenghi L, Maier JAM, Bradamante S. The challenging environment on board the International Space Station affects endothelial cell function by triggering oxidative stress through thioredoxin interacting protein overexpression: the ESA-SPHINX experiment. FASEB Journal. 2013;27(11):4466-4475. doi:10.1096/fj.13-229195	cell culture	gravity exposure	trancriptomics	
Italy	Vigorelli V, Resta J, Bianchessi V, Lauri A, Bassetti B, Agrifoglio M, Pesce M, Polvani G, Bonalumi G, Cavallotti L, Alamanni F, Genovese S, Pompilio G, Vinci MC. Abnormal DNA Methylation Induced by Hyperglycemia Reduces CXCR 4 Gene Expression in CD 34+Stem	cell culture		trancriptomics; epigenetics	

	Cells. J Am Heart Assoc. 2019 May 7;8(9):e010012. doi: 10.1161/JAHA.118.010012. PMID: 31018749; PMCID: PMC6512087.				
Italy	Villa I, Senesi P, Montesano A, Ferraretto A, Vacante F, Spinello A, Bottani M, Bolamperti S, Rubinacci A, Luzi L, Terruzzi I. Betaine promotes cell differentiation of human osteoblasts in primary culture. J Transl Med. 2017 Jun 7;15(1):132. doi: 10.1186/s12967-017-1233-5. PMID: 28592272; PMCID: PMC5463390.	cell culture		administration assay	
Italy	Villani ER, Onder G, Carfì A, Di Segni C, Raimondo S, Silvestrini A, Meucci E, Mancini A. Thyroid Function and its Implications in Oxidative Stress Influencing the Pathogenesis of Osteoporosis in Adults with Down Syndrome: A Cohort Study. Horm Metab Res. 2016 Sep;48(9):565-70. doi: 10.1055/s-0042-112127. Epub 2016 Aug 24. PMID: 27557341.		Down syndrome		
Japan	Ando W, Tateishi K, Katakai D, Hart DA, Higuchi C, Nakata K, Hashimoto J, Fujie H, Shino K, Yoshikawa H, Nakamura N. In vitro generation of a scaffold-free tissue-engineered construct (TEC) derived from human synovial mesenchymal stem cells: biological and mechanical properties and further chondrogenic potential. Tissue Eng Part A. 2008 Dec;14(12):2041-9. doi: 10.1089/ten.tea.2008.0015. PMID: 18636944.	cell culture		functional assay	
Japan	Eiko Sakai, Masanobu Morita, Masahiro Ohuchi, et al. Effects of deficiency of Kelch-like ECH-associated protein 1 on skeletal organization: a mechanism for diminished nuclear factor of activated T cells cytoplasmic 1 during osteoclastogenesis. FASEB Journal. 2017;31(9):4011-4022. doi:10.1096/fj.201700177R	animals		transcriptomics	
Japan	Liu L, Igarashi K, Kanzaki H, Chiba M, Shinoda H, Mitani H. Clodronate inhibits PGE(2) production in compressed periodontal ligament cells. J Dent Res. 2006 Aug;85(8):757-60. doi: 10.1177/154405910608500813. PMID: 16861295.	cell culture	periodontitis	administration assay	
Japan	Miura S, Yamaguchi M, Yoshino H, Nakai Y, Kashiwakura I. Dose-Dependent Increase of Nrf2 Target Gene Expression in Mice Exposed to Ionizing Radiation. Radiation Research. 2019;191(2):176-188. doi:10.1667/RR15203.1	animals		transcriptomics ; administration assay	
Japan	Mori K, Kitazawa R, Kondo T, et al. Diabetic Osteopenia by Decreased β-Catenin Signaling Is Partly Induced by Epigenetic Derepression of sFRP-4 Gene. PLoS ONE. 2014;9(7):1-11. doi:10.1371/journal.pone.0102797	cell culture		epigenetics	
Japan	Nagaoka M, Maeda T, Moriwaki S, et al. Petunidin, a B-ring 5′-O-Methylated Derivative of Delphinidin, Stimulates Osteoblastogenesis and Reduces sRANKL-Induced Bone Loss. International Journal of Molecular Sciences. 2019;20(11):2795. doi:10.3390/ijms20112795	cell culture		administration assay	
Japan	Nakai, S., M. Fujita, and Y. Kamei. "Health Promotion Effects of Soy Isoflavones." Journal of Nutritional Science and Vitaminology, vol. 66, no. 6, 2020, pp. 502-507. SCOPUS, www.scopus.com, doi:10.3177/jnsv.66.502.			diet supplements	review
Japan	Nishioku T, Kawamoto M, Okizono R, Sakai E, Okamoto K, Tsukuba T. Dimethyl fumarate prevents osteoclastogenesis by decreasing NFATc1 expression, inhibiting of erk and p38 MAPK phosphorylation, and suppressing of HMGB1 release. Biochemical & Biophysical Research Communications. 2020;530(2):455-461. doi:10.1016/j.bbrc.2020.05.088	cell culture		administration assay	
Japan	Satomura K, Tobiume S, Tokuyama R, Yamasaki Y, Kudoh K, Maeda E, Nagayama M. Melatonin at pharmacological doses enhances human osteoblastic differentiation in vitro and promotes mouse cortical bone formation in vivo. J Pineal Res. 2007 Apr;42(3):231-9. doi: 10.1111/j.1600-079X.2006.00410.x. PMID: 17349020.	animals		administration assay	

Japan	Sogi, Y., Yabe, Y., Hagiwara, Y., Tsuchiya, M., Onoda, Y., Sekiguchi, T., Itaya, N., Yoshida, S., Yano, T., Suzuki, K., Onoki, T. & Itoi, E. (2020). Joint hemorrhage accelerates cartilage degeneration in a rat immobilized knee model. BMC Musculoskeletal Disorders, 21(1). https://doi.org/10.1186/s12891-020-03795-0	animals	functional assay	
Japan	Wada, S., et al. "Bach1 Inhibition Suppresses Osteoclastogenesis Via Reduction of the Signaling Via Reactive Oxygen Species by Reinforced Antioxidation." Frontiers in Cell and Developmental Biology, vol. 8, 2020. SCOPUS, www.scopus.com, doi:10.3389/fcell.2020.00740.	cell culture	functional assay	
Japan	Yamaguchi M. Role of Regucalcin in Cell Homeostasis and Disorder. Nova Science Publishers, Inc; 2017. Accessed November 5, 2022. https://search.ebscohost.com/login.aspx?direct=true&AuthType=sso&db=nlebk&AN=1440447&lang=es&site=ehost-live		compound administration	book
Korea	Kim E-N, Kim G-R, Yu JS, Kim KH, Jeong G-S. Inhibitory Effect of (2 R)-4-(4-hydroxyphenyl)-2-butanol 2- O -β-d-apiofuranosyl-(1→6)-β-d-glucopyranoside on RANKL-Induced Osteoclast Differentiation and ROS Generation in Macrophages. International Journal of Molecular Sciences. 2021;22(1):222. doi:10.3390/ijms22010222	cell culture	administration assay	
Korea	Park E, Lee CG, Jeong H, et al. Antiadipogenic Effects of Mixtures of Cornus officinalis and Ribes fasciculatum Extracts on 3T3-L1 Preadipocytes and High-Fat Diet-Induced Mice. Molecules. 2020;25(10):2350. doi:10.3390/molecules25102350	animals; cell culture	functional assay	
Korea	Park, K. H., et al. "Anti-Osteoporosis Effects of the Fruit of Sea Buckthorn (Hippophae Rhamnoides) through Promotion of Osteogenic Differentiation in Ovariectomized Mice." Nutrients, vol. 14, no. 17, 2022. SCOPUS, www.scopus.com, doi:10.3390/nu14173604.	animals	administration assay	
Latvia	Saulite L, Jekabsons K, Klavins M, Muceniece R, Riekstina U. Effects of malvidin, cyanidin and delphinidin on human adipose mesenchymal stem cell differentiation into adipocytes, chondrocytes and osteocytes. Phytomedicine. 2019 Feb;53:86-95. doi: 10.1016/j.phymed.2018.09.029. Epub 2018 Sep 5. PMID: 30668416.	cell culture	administration assay	
Malaysia	Ramli, E., Ahmad, F., Salleh, N., Ahmed, A., Hee, C., Kumar, S., Abdullah, M., Yahaya, F. & Soelaiman, I. (2018). Beneficial Effects of Annatto (Bixa Orellana) Tocotrienol on Bone Histomorphometry and Expression of Genes Related to Bone Formation and Resorption in Osteoporosis Induced by Dexamethasone. INTERNATIONAL JOURNAL OF MEDICAL RESEARCH & HEALTH SCIENCES, 7(12), 85-100. https://www-webofscience-com.pbidi.unam.mx:2443/wos/woscc/full-record/WOS:000457008900014	animals	administration assay	
Malaysia	Regulation of Gene Expressions. Medicine & Health (Universiti Kebangsaan Malaysia). 2018;13(1):175-197. doi:10.17576/MH.2018.1301.17	animals	administration assay	
Malaysia	Sridevi, V., et al. "Beneficiary and Adverse Effects of Phytoestrogens: A Potential Constituent of Plant-Based Diet." Current Pharmaceutical Design, vol. 27, no. 6, 2021, pp. 802-815. SCOPUS, www.scopus.com, doi:10.2174/1381612826999200917154747.			review
Malaysia	Wong, S. K., et al. "Potential Role of Tocotrienols on Non-Communicable Diseases: A Review of Current Evidence." Nutrients, vol. 12, no. 1, 2020. SCOPUS, www.scopus.com, doi:10.3390/nu12010259.			review
Netherlands	Alves RD, Demmers JA, Bezstarosti K, van der Eerden BC, Verhaar JA, Eijken M, van Leeuwen JP. Unraveling the human bone microenvironment beyond the classical extracellular		proteomics	

	matrix proteins: a human bone protein library. J Proteome Res. 2011 Oct 7;10(10):4725-33. doi: 10.1021/pr200522n. Epub 2011 Sep 21. Erratum in: J Proteome Res. 2011 Dec 2;10(2):5576. PMID: 21892838.				
Netherlands	Saeed A, Hoogerland JA, Wessel H, Heegsma J, Derks TGJ, van der Veer E, Mithieux G, Rajas F, Oosterveer MH, Faber KN. Glycogen storage disease type 1a is associated with disturbed vitamin A metabolism and elevated serum retinol levels. Hum Mol Genet. 2020 Jan 15;29(2):264-273. doi: 10.1093/hmg/ddz283. PMID: 31813960; PMCID: PMC7001719.	animals		functional assay	
New Zealand	Scott, L. J. "Repaglinide: A Review of its use in Type 2 Diabetes Mellitus." Drugs, vol. 72, no. 2, 2012, pp. 249-272. SCOPUS, www.scopus.com, doi:10.2165/11207600-000000000-00000.				review
Oman	Sampat N, Al-Balushi B, Al-Subhi L, Al-Adawi S, Essa M, Walid Qoronfleh M. Vitamin D: Public Health Status Regional Gulf Region. International Journal of Nutrition, Pharmacology, Neurological Diseases. 2019;9(4):117-135. doi:10.4103/ijnpnd.ijnpnd_68_19				review
Poland	Brzęczek M, Hyla-Klekot L, Kokot F, Synder M. Contribution of Bone Tissue to Regulation of Calcium and Phosphate Metabolism. Role of FGF23 and Klotho Protein. Ortop Traumatol Rehabil. 2020 Apr 30;22(2):69-76. doi: 10.5604/01.3001.0014.1153. PMID: 32468993.				review
Poland	Mydel P, Takahashi Y, Yumoto H, et al. Roles of the Host Oxidative Immune Response and Bacterial Antioxidant Rubrerythrin during Porphyromonas gingivalis Infection. PLoS Pathogens. 2006;2(7):e76-0725. doi:10.1371/journal.ppat.0020076	animals	gingivitis		
Poland	Zakłos-Szyda M, Nowak A, Pietrzyk N, Podsędek A. Viburnum opulus L. Juice Phenolic Compounds Influence Osteogenic Differentiation in Human Osteosarcoma Saos-2 Cells. International Journal of Molecular Sciences. 2020;21(14):4909. doi:10.3390/ijms21144909	cell culture		administration assay	
Portugal	Costa-Rodrigues J, Fernandes MH, Pinho O, Monteiro PRR. Modulation of human osteoclastogenesis and osteoblastogenesis by lycopene. J Nutr Biochem. 2018 Jul;57:26-34. doi: 10.1016/j.jnutbio.2018.03.004. Epub 2018 Mar 15. PMID: 29655028.	cell culture		administration assay	
Portugal	Costa-Rodrigues, J., et al. "Modulation of Human Osteoclastogenesis and Osteoblastogenesis by Lycopene." Journal of Nutritional Biochemistry, vol. 57, 2018, pp. 26-34. SCOPUS, www.scopus.com, doi:10.1016/j.jnutbio.2018.03.004.	cell culture		functional assay	
Portugal	Dias, J. S. "Vegetable Breeding for Nutritional Quality and Health Benefits." Cultivars: Chemical Properties, Antioxidant Activities and Health Benefits. , 2013. SCOPUS, www.scopus.com.			diet supplements	review
Portugal	João Costa-Rodrigues, Maria Helena Fernandes, Olívia Pinho, Pedro Ribeiro Rocha Monteiro, Modulation of human osteoclastogenesis and osteoblastogenesis by lycopene, The Journal of Nutritional Biochemistry, Volume 57, 2018, Pages 26-34, ISSN 0955-2863, https://doi.org/10.1016/j.jnutbio.2018.03.004.(https://www.sciencedirect.com/science/article/pii/S0955286317304849)	cell culture		functional assay	
South Korea	Chung IJ, Lee JJ, Nam CE, Kim HN, Kim YK, Park MR, Cho SH, Kim HJ. Increased inducible nitric oxide synthase expression and nitric oxide concentration in patients with aplastic anemia. Ann Hematol. 2003 Feb;82(2):104-108. doi: 10.1007/s00277-002-0602-0. Epub 2003 Jan 30. PMID: 12601489.		anaemia	transcriptomics	
Romania	Domsa, E. -., et al. "Celiac Disease: A Multi-Faceted Medical Condition." Journal of Physiology and Pharmacology, vol. 71, no. 1, 2020, pp. 1-12. SCOPUS, www.scopus.com, doi:10.26402/jpp.2020.1.01.				review

Slovenia	Trošt Z, Trebše R, Preželj J, Komadina R, Logar DB, Marc J. A microarray based identification of osteoporosis-related genes in primary culture of human osteoblasts. BONE. 2010;46(1):72-80. doi:10.1016/j.bone.2009.09.015	cell culture		transcriptomics	
Slovenia	Vrtačnik P, Zupan J, Mlakar V, et al. Epigenetic enzymes influenced by oxidative stress and hypoxia mimetic in osteoblasts are differentially expressed in patients with osteoporosis and osteoarthritis. Scientific Reports. 2018;8(1):1. doi:10.1038/s41598-018-34255-4	cell culture		transcriptomics ; epigenetics	
South Korea	Kim M, Lee YJ, Jee SC, Choi I, Sung JS. Anti-adipogenic effects of sesamol on human mesenchymal stem cells. Biochem Biophys Res Commun. 2016 Jan 1;469(1):49-54. doi: 10.1016/j.bbrc.2015.11.070. Epub 2015 Nov 23. PMID: 26616060.	cell culture	adiposity	administration assay	
South Korea	Kim MH, Ryu SY, Bae MA, Choi JS, Min YK, Kim SH. Baicalein inhibits osteoclast differentiation and induces mature osteoclast apoptosis. Food Chem Toxicol. 2008 Nov;46(11):3375-82. doi: 10.1016/j.fct.2008.08.016. Epub 2008 Aug 26. PMID: 18786594.	cell culture		administration assay	
South Korea	Lee W, Ko KR, Kim H, Lim S, Kim S. Dehydrodiconiferyl alcohol promotes BMP-2-induced osteoblastogenesis through its agonistic effects on estrogen receptor. Biochemical & Biophysical Research Communications. 2018;495(3):2242-2248. doi:10.1016/j.bbrc.2017.12.079	cell culture		administration assay	
Spain	Bullon P, Battino M, Varela-Lopez A, et al. Diets Based on Virgin Olive Oil or Fish Oil but Not on Sunflower Oil Prevent Age-Related Alveolar Bone Resorption by Mitochondrial-Related Mechanisms. PLoS ONE. 2013;8(9):1. doi:10.1371/journal.pone.0074234	animals		transcriptomics : diet assay	
Spain	Casado-Díaz A, Anter J, Dorado G, Quesada-Gómez JM. Effects of quercetin, a natural phenolic compound, in the differentiation of human mesenchymal stem cells (MSC) into adipocytes and osteoblasts. J Nutr Biochem. 2016 Jun;32:151-62. doi: 10.1016/j.jnutbio.2016.03.005. Epub 2016 Mar 30. PMID: 27142748.	cell culture		administration assay	
Spain	Pineda, B., et al. "Gene Expression Profile Induced by Ovariectomy in Bone Marrow of Mice: A Functional Approach to Identify New Candidate Genes Associated to Osteoporosis Risk in Women." Bone, vol. 65, 2014, pp. 33-41. SCOPUS, www.scopus.com, doi:10.1016/j.bone.2014.05.001.	animals		trascriptomics	
Spain	Portal-Núñez S, Manassra R, Lozano D, Acitores A, Mulero F, Villanueva-Peñacarrillo ML, De la Fuente M, Esbrit P. Characterization of skeletal alterations in a model of prematurely aging mice. Age (Dordr). 2013 Apr;35(2):383-93. doi: 10.1007/s11357-011-9372-8. Epub 2012 Jan 11. PMID: 22234865; PMCID: PMC3592965.	animals		functional assay	
Spain	Román-Malo L, Bullon P. Influence of the Periodontal Disease, the Most Prevalent Inflammatory Event, in Peroxisome Proliferator-Activated Receptors Linking Nutrition and Energy Metabolism. International Journal of Molecular Sciences. 2017;18(7):1438. doi:10.3390/ijms18071438		periodontitis		review
Spain	Tresguerres JÁF, Fernández-Tresguerres I, Viña J, Rancan L, Paredes SD, Linillos-Pradillo B, Vara E. Effects of GH on the Aging Process in Several Organs: Mechanisms of Action. Int J Mol Sci. 2022 Jul 16;23(14):7848. doi: 10.3390/ijms23147848. PMID: 35887196; PMCID: PMC9318627.	animals		adminsitration assay	
Sweden	Söderquist F, Janson ET, Rasmusson AJ, Ali A, Stridsberg M, Cunningham JL. Melatonin Immunoreactivity in Malignant Small Intestinal Neuroendocrine Tumours. PLoS One. 2016 Oct	cell culture	tumor	adminsitration assay	

	13;11(10):e0164354. doi: 10.1371/journal.pone.0164354. PMID: 27736994; PMCID: PMC5063280.				
Taiwan	Hsieh TP, Sheu SY, Sun JS, Chen MH, Liu MH. Icariin isolated from Epimedium pubescens regulates osteoblasts anabolism through BMP-2, SMAD4, and Cbfa1 expression. Phytomedicine. 2010 May;17(6):414-23. doi: 10.1016/j.phymed.2009.08.007. Epub 2009 Sep 10. PMID: 19747809.	cell culture		functional assay	
Thailand	Roomruangwong, C., and M. Maes. "Biomarker Validation of a New Case Definition of Menstrual Cycle-Associated Syndrome (MCAS) Opinion Paper." CNS and Neurological Disorders - Drug Targets, vol. 20, no. 2, 2021, pp. 105-111. SCOPUS, www.scopus.com, doi:10.2174/1871527319666200930095149.				review; opinion paper
The Netherlands	Nicolaije C, Diderich KEM, Botter SM, et al. Age-Related Skeletal Dynamics and Decrease in Bone Strength in DNA Repair Deficient Male Trichothiodystrophy Mice. PLoS ONE. 2012;7(4):1-13. doi:10.1371/journal.pone.0035246	animals		functional assay	
Turkey	Aydin H, Deyneli O, Yavuz D, Yüksel M, Tarçin O, Yazici D, Tutepe H, Akalin S. Effect of oxidative stress on aorta and tibia osteoprotegerin gene expression in ovariectomized rats. Minerva Endocrinol. 2011 Jun;36(2):107-15. PMID: 21519319.	animals		transcriptomics	
Turkey	Kara M, Boran T, Öztaş E, Jannuzzi AT, Özden S, Özhan G. Zoledronic acid-induced oxidative damage and endoplasmic reticulum stress-mediated apoptosis in human embryonic kidney (HEK-293) cells. Journal of Biochemical & Molecular Toxicology. 2022;36(8):1-14. doi:10.1002/jbt.23083	cell culture		functional assay	
UK	Kirk B, Kuo CL, Xiang M, Duque G. Associations between leukocyte telomere length and osteosarcopenia in 20,400 adults aged 60 years and over: Data from the UK Biobank. Bone. 2022 Aug;161:116425. doi: 10.1016/j.bone.2022.116425. Epub 2022 Apr 27. PMID: 35489708.			telomeres	
UK	Malde, S., R. Cartwright, and K. A. O. Tikkinen. "What's New in Epidemiology?" European Urology Focus, vol. 4, no. 1, 2018, pp. 11-13. SCOPUS, www.scopus.com, doi:10.1016/j.euf.2018.02.003.				review
UK	Phillipson OT. Alpha-synuclein, epigenetics, mitochondria, metabolism, calcium traffic, & circadian dysfunction in Parkinson's disease. An integrated strategy for management. Ageing Research Reviews. 2017;40:149-167. doi:10.1016/j.arr.2017.09.006		Parkinson		review
UK	Varanasi SS, Datta HK. Southern analysis of mitochondrial DNA in cortical bone of elderly patients undergoing knee and hip arthroplasty. J Pathol. 2001 Apr;193(4):557-62. doi: 10.1002/path.823. PMID: 11276017.			mitochondria	
USA	Almeida M, Ambrogini E, Han L, Manolagas SC, JiIka RL. Increased Lipid Oxidation Causes Oxidative Stress, Increased Peroxisome Proliferator-activated Receptor-γ Expression, and Diminished Pro-osteogenic Wnt Signaling in the Skeleton. Journal of Biological Chemistry. 2009;284(40):27438-27448. doi:10.1074/jbc.M109.023572	animals		administration assay	
USA	Almeida M, Li Han, Martin-Millan M, O'Brien CA, Manolagas SC. Oxidative Stress Antagonizes Wnt Signaling in Osteoblast Precursors by Diverting β-Catenin from T Cell Factor- to Forkhead Box O-mediated Transcription. Journal of Biological Chemistry. 2007;282(37):27298-27305. doi:10.1074/jbc.M702811200	animals		transcriptomics ; administration assay	

USA	Almeida, M. S. (2009b). The Basic Biology of Estrogen and Bone. Osteoporosis, 333-350. https://doi.org/10.1007/978-1-59745-459-9_14				review
USA	Ariza, M. E. "Myalgic encephalomyelitis/chronic Fatigue Syndrome: The Human Herpesviruses are Back!" Biomolecules, vol. 11, no. 2, 2021, pp. 1-17. SCOPUS, www.scopus.com, doi:10.3390/biom11020185.				review
USA	Bhatti, U. F., et al. "Assessment of the Cytoprotective Effects of High-Dose Valproic Acid Compared to a Clinically used Lower Dose." Journal of Surgical Research, vol. 266, 2021, pp. 125-141. SCOPUS, www.scopus.com, doi:10.1016/j.jss.2021.03.025		periodontitis	administration assay	
USA	Bollag AE, Guo T, Ding KH, Choudhary V, Chen X, Zhong Q, Xu J, Yu K, Awad ME, Elsalanty M, Johnson MH, McGee-Lawrence ME, Bollag WB, Isales CM. Monomethylfumarate protects against ovariectomy-related changes in body composition. J Endocrinol. 2019 Jul 1:JOE-18-0691.R3. doi: 10.1530/JOE-18-0691. Epub ahead of print. PMID: 31362266; PMCID: PMC6938560.	animals		administration assay	
USA	Boregowda SV, Ghoshal S, Booker CN, Krishnappa V, Chakraborty A, Phinney DG. IP6K1 Reduces Mesenchymal Stem/Stromal Cell Fitness and Potentiates High Fat Diet-Induced Skeletal Involution. Stem Cells. 2017 Aug;35(8):1973-1983. doi: 10.1002/stem.2645. Epub 2017 Jun 15. PMID: 28577302; PMCID: PMC5533188.	animals		functional assay	
USA	Chandra A, Lagnado AB, Farr JN, et al. Bone Marrow Adiposity in Models of Radiation- and Aging-Related Bone Loss Is Dependent on Cellular Senescence. Journal of Bone & Mineral Research. 2022;37(5):997-1011. Accessed November 5, 2022. https://search.ebscohost.com/login.aspx?direct=true&AuthType=sso&db=s3h&AN=156806075&lang=es&site=ehost-live	cell culture		transcriptomics	
USA	Davies JMS, Cillard J, Friguet B, Cadenas E, Cadet J, Cayce R, Fishmann A, Liao D, Bulteau AL, Derbré F, Rébillard A, Burstein S, Hirsch E, Kloner RA, Jakowec M, Petzinger G, Sauce D, Sennlaub F, Limon I, Ursini F, Maiorino M, Economides C, Pike CJ, Cohen P, Salvayre AN, Halliday MR, Lundquist AJ, Jakowec NA, Mechta-Grigoriou F, Mericskay M, Mariani J, Li Z, Huang D, Grant E, Forman HJ, Finch CE, Sun PY, Pomatto LCD, Agbulut O, Warburton D, Neri C, Rouis M, Cillard P, Capeau J, Rosenbaum J, Davies KJA. The Oxygen Paradox, the French Paradox, and age-related diseases. Geroscience. 2017 Dec;39(5-6):499-550. doi: 10.1007/s11357-017-0002-y. Epub 2017 Dec 21. PMID: 29270905; PMCID: PMC5745211.				review
USA	Douvas, M. G., and L. L. Riegler. "Meeting Challenges in the Long-Term Care of Children, Adolescents, and Young Adults with Acute Lymphoblastic Leukemia." Current Hematologic Malignancy Reports, vol. 17, no. 1, 2022, pp. 15-24. SCOPUS, www.scopus.com, doi:10.1007/s11899-021-00657-x.				review
USA	Elis S, Wu Y, Courtland H-W, et al. Increased serum IGF-1 levels protect the musculoskeletal system but are associated with elevated oxidative stress markers and increased mortality independent of tissue igf1 gene expression. Aging Cell. 2011;10(3):547-550. doi:10.1111/j.1474-9726.2011.00683.x	animals			
USA	Fargaly H, Mathew S, Rossi NF. Hyperglycinuria: diagnosis in middle age. BMJ Case Rep. 2022 Mar 2;15(3):e246252. doi: 10.1136/bcr-2021-246252. PMID: 35236679; PMCID: PMC8895892.				case report; review

USA	Fariyike B, Singleton Q, Hunter M, et al. Role of MicroRNA-141 in the Aging Musculoskeletal System: A Current Overview. Mechanisms of Ageing & Development. 2019;178:9-15. doi:10.1016/j.mad.2018.12.001				review
USA	Finkelstein, Y., et al. "A Thymidylate Synthase Polymorphism is Associated with Increased Risk for Bone Toxicity among Children Treated for Acute Lymphoblastic Leukemia." Pediatric Blood and Cancer, vol. 64, no. 7, 2017. SCOPUS, www.scopus.com, doi:10.1002/pbc.26393.	children			
USA	Gan X, Huang S, Yu Q, Yu H, Yan SS. Blockade of Drp1 rescues oxidative stress-induced osteoblast dysfunction. Biochemical & Biophysical Research Communications. 2015;468(4):719-725. doi:10.1016/j.bbrc.2015.11.022	cell culture		functional assay	
USA	Garimella R, Tadikonda P, Tawfik O, Gunewardena S, Rowe P, Van Veldhuizen P. Vitamin D Impacts the Expression of Runx2 Target Genes and Modulates Inflammation, Oxidative Stress and Membrane Vesicle Biogenesis Gene Networks in 143B Osteosarcoma Cells. Int J Mol Sci. 2017 Mar 16;18(3):642. doi: 10.3390/ijms18030642. PMID: 28300755; PMCID: PMC5372654.	cell culture		transcriptomics	
USA	Gong Z, Kennedy O, Sun H, et al. Reductions in serum IGF-1 during aging impair health span. Aging Cell. 2014;13(3):408-418. doi:10.1111/acel.12188	animals			
USA	Guo, T. -., et al. "Oxidative Stress Contributes to Fracture/Cast-Induced Inflammation and Pain in a Rat Model of Complex Regional Pain Syndrome." Journal of Pain, vol. 19, no. 10, 2018, pp. 1147-1156. SCOPUS, www.scopus.com, doi:10.1016/j.jpain.2018.04.006.	animals		functional assay	
USA	Kakoki M, Kizer CM, Xianwen Yi, et al. Senescence-associated phenotypes in Akita diabetic mice are enhanced by absence of bradykinin B2 receptors. Journal of Clinical Investigation. 2006;116(5):1302-1309. doi:10.1172/JCI26958	animals			
USA	Kakokia M, Sullivan KA, Backus C, et al. Lack of both bradykinin B1 and B2 receptors enhances nephropathy, neuropathy, and bone mineral loss in Akita diabetic mice. Proceedings of the National Academy of Sciences of the United States of America. 2010;107(22):10190-10195. doi:10.1073/pnas.1005144107	animals			
USA	Kalyanaraman H, Ramdani G, Joshua J, et al. A Novel, Direct NO Donor Regulates Osteoblast and Osteoclast Functions and Increases Bone Mass in Ovariectomized Mice. Journal of Bone & Mineral Research. 2017;32(1):46-59. Accessed November 5, 2022. https://search.ebscohost.com/login.aspx?direct=true&AuthType=sso&db=s3h&AN=120631085&lang=es&site=ehost-live	animals		functional assay	
USA	Kalyanaraman H, Schwaerzer G, Ramdani G, et al. Protein Kinase G Activation Reverses Oxidative Stress and Restores Osteoblast Function and Bone Formation in Male Mice With Type 1 Diabetes. Diabetes. 2018;67(4):607-623. doi:10.2337/db17-0965	animals	diabetes	administration assay	
USA	Levin, E. R. "Translating Extranuclear Steroid Receptor Signaling to Clinical Medicine." Hormones and Cancer, vol. 5, no. 3, 2014, pp. 140-145. SCOPUS, www.scopus.com, doi:10.1007/s12672-014-0179-9.				review
USA	Li X, Jiang M, Tan T, Narasimhulu CA, Xiao Y, Hao H, Cui Y, Zhang J, Liu L, Yang C, Li Y, Ma J, Verfaillie CM, Parthasarathy S, Zhu H, Liu Z. N-acetylcysteine prevents oxidized low-density lipoprotein-induced reduction of MG53 and enhances MG53 protective effect on bone marrow stem cells. J Cell Mol Med. 2020 Jan;24(1):886-898. doi: 10.1111/jcmm.14798. Epub 2019 Nov 19. PMID: 31742908; PMCID: PMC6933383.	cell culture		functional assay	

USA	Liang C, Oest ME, Jones JC, Prater MR. Gestational high saturated fat diet alters C57BL/6 mouse perinatal skeletal formation. Birth Defects Res B Dev Reprod Toxicol. 2009 Oct;86(5):362-9. doi: 10.1002/bdrb.20204. PMID: 19750487.	animals	diet intervention
USA	Liu, Z., et al. "Mitochondrial Function is Compromised in Cortical Bone Osteocytes of Long-Lived Growth Hormone Receptor Null Mice." Journal of Bone and Mineral Research, vol. 34, no. 1, 2019, pp. 106-122. SCOPUS, www.scopus.com, doi:10.1002/jbmr.3573.	animals	mitochondria
USA	Manavalan JS, Cremers S, Dempster DW, Zhou H, Dworakowski E, Kode A, Kousteni S, Rubin MR. Circulating osteogenic precursor cells in type 2 diabetes mellitus. J Clin Endocrinol Metab. 2012 Sep;97(9):3240-50. doi: 10.1210/jc.2012-1546. Epub 2012 Jun 27. PMID: 22740707; PMCID: PMC3431571.	cell culture	
USA	Martin, S. A., et al. "Rapamycin Impairs Bone Accrual in Young Adult Mice Independent of Nrf2." Experimental Gerontology, vol. 154, 2021. SCOPUS, www.scopus.com, doi:10.1016/j.exger.2021.111516	animals	administration assay
USA	Meyer MH, Meyer RA Jr. Altered expression of mitochondrial genes in response to fracture in old rats. Acta Orthop. 2006 Dec;77(6):944-51. doi: 10.1080/17453670610013277. PMID: 17260206.	animals	transcriptomics
USA	O'Sullivan RP, Greenberger JS, Goff J, et al. Dysregulated in vitro hematopoiesis, radiosensitivity, proliferation, and osteoblastogenesis with marrow from SAMP6 mice. Experimental Hematology. 2012;40(6):499-509. doi:10.1016/j.exphem.2012.01.019	cell culture	administration assay
USA	Onal M, Piemontese M, Xiong J, Wang Y, Han L, Ye S, Komatsu M, Selig M, Weinstein RS, Zhao H, Jilka RL, Almeida M, Manolagas SC, O'Brien CA. Suppression of autophagy in osteocytes mimics skeletal aging. J Biol Chem. 2013 Jun 14;288(24):17432-40. doi: 10.1074/jbc.M112.444190. Epub 2013 May 3. PMID: 23645674; PMCID: PMC3682543.	animals	functional assay
USA	Palmieri M, Almeida M, Nookaew I, Gomez-Acevedo H, Joseph TE, Que X, Tsimikas S, Sun X, Manolagas SC, Witztum JL, Ambrogini E. Neutralization of oxidized phospholipids attenuates age-associated bone loss in mice. Aging Cell. 2021 Aug;20(8):e13442. doi: 10.1111/acel.13442. Epub 2021 Jul 19. PMID: 34278710; PMCID: PMC8373359.	animals	functional assay
USA	Park J, Wick HC, Kee DE, Noto K, Maron JL, Slonim DK. Finding novel molecular connections between developmental processes and disease. PLoS Comput Biol. 2014 May 29;10(5):e1003578. doi: 10.1371/journal.pcbi.1003578. PMID: 24874013; PMCID: PMC4038461.		web-based visualization tool
USA	Pedersen KB, Osborn ML, Robertson AC, Williams AE, Watt J, Denys A, Schröder K, Ronis MJ. Chronic Ethanol Feeding in Mice Decreases Expression of Genes for Major Structural Bone Proteins in a Nox4-Independent Manner. J Pharmacol Exp Ther. 2020 Jun;373(3):337-346. doi: 10.1124/jpet.119.264374. Epub 2020 Mar 25. PMID: 32213546; PMCID: PMC7228502.	animals	transcriptomics ; administration assay
USA	Pellegrini GG, Cregor M, McAndrews K, Morales CC, McCabe LD, McCabe GP, Peacock M, Burr D, Weaver C, Bellido T. Nrf2 regulates mass accrual and the antioxidant endogenous response in bone differently depending on the sex and age. PLoS One. 2017 Feb 2;12(2):e0171161. doi: 10.1371/journal.pone.0171161. PMID: 28152064; PMCID: PMC5289572.	animals	functional assay

USA	Pennisi A, Wen Ling, Xin Li, et al. Consequences of Daily Administered Parathyroid Hormone on Myeloma Growth, Bone Disease, and Molecular Profiling of Whole Myelomatous Bone. PLoS ONE. 2010;5(12):1-13. doi:10.1371/journal.pone.0015233	cell culture	transcriptomics ; administration assay	
USA	Posey KL, Coustry F, Hecht JT. Cartilage oligomeric matrix protein: COMPopathies and beyond. Matrix Biology. 2018;71:161-173. doi:10.1016/j.matbio.2018.02.023			review
USA	Prater, M. R. "Nutritional Aspects Relating to the Developmental Origins of Health and Disease." Current Women's Health Reviews, vol. 4, no. 3, 2008, pp. 143-152. SCOPUS, www.scopus.com, doi:10.2174/157340408785821827.			review
USA	Prisby RD, Ramsey MW, Behnke BJ, Dominguez JM 2nd, Donato AJ, Allen MR, Delp MD. Aging reduces skeletal blood flow, endothelium-dependent vasodilation, and NO bioavailability in rats. J Bone Miner Res. 2007 Aug;22(8):1280-8. doi: 10.1359/jbmr.070415. PMID: 17451371.	animals	functional assay	
USA	Qian D, Zhou H, Fan P, Yu T, Patel A, O'Brien M, Wang Z, Lu S, Tong G, Shan Y, Wang L, Gao Y, Xiong Y, Zhang L, Wang X, Liu Y, Zhou S. A Traditional Chinese Medicine Plant Extract Prevents Alcohol-Induced Osteopenia. Front Pharmacol. 2021 Dec 15;12:754088. doi: 10.3389/fphar.2021.754088. PMID: 35002697; PMCID: PMC8730326.	animals	administration assay	
USA	Rana T, Schultz MA, Freeman ML, Biswas S. Loss of Nrf2 accelerates ionizing radiation-induced bone loss by upregulating RANKL. Free Radical Biology & Medicine. 2012;53(12):2298-2307. doi:10.1016/j.freeradbiomed.2012.10.536	animals	administration assay	
USA	Saleh MA, McMaster WG, Jing Wu, et al. Lymphocyte adaptor protein LNK deficiency exacerbates hypertension and end-organ inflammation. Journal of Clinical Investigation. 2015;125(3):1189-1202. doi:10.1172/JCI76327	animals		
USA	Savage SA, Alter BP. The role of telomere biology in bone marrow failure and other disorders. Mechanisms of Ageing & Development. 2008;129(1/2):35-47. doi:10.1016/j.mad.2007.11.002		telomeres	review
USA	Smith BJ, Graef JL, Wronski TJ, Rendina E, Williams AA, Clark KA, Clarke SL, Lucas EA, Halloran BP. Effects of dried plum supplementation on bone metabolism in adult C57BL/6 male mice. Calcif Tissue Int. 2014 Apr;94(4):442-53. doi: 10.1007/s00223-013-9819-2. Epub 2013 Dec 20. PMID: 24357047; PMCID: PMC3950615.	animals	administration assay	
USA	Smith RL, Lindsey DP, Dhulipala L, Harris AH, Goodman SB, Maloney WJ. Effects of intermittent hydrostatic pressure and BMP-2 on osteoarthritic human chondrocyte metabolism in vitro. J Orthop Res. 2011 Mar;29(3):361-8. doi: 10.1002/jor.21250. Epub 2010 Sep 29. PMID: 20882590.	cell culture	functional assay	
USA	Stark WS, White RH. Carotenoid replacement in Drosophila: freeze-fracture electron microscopy. J Neurocytol. 1996 Apr;25(4):233-41. doi: 10.1007/BF02284799. PMID: 8793729.	animals	administration assay	
USA	Tilstra JS, Clauson CL, Niedernhofer LJ, Robbins PD. NF-κB in Aging and Disease. Aging & Disease. 2011;2(6):449-465. Accessed November 5, 2022. https://search.ebscohost.com/login.aspx?direct=true&AuthType=sso&db=a9h&AN=69883026&lang=es&site=ehost-live			review
USA	Tower, J. "Sex-Specific Gene Expression and Life Span Regulation." Trends in Endocrinology and Metabolism, vol. 28, no. 10, 2017, pp. 735-747. SCOPUS, www.scopus.com, doi:10.1016/j.tem.2017.07.002.		transcriptomics	review

USA	Vasiliou V, Thompson DC, Smith C, Fujita M, Chen Y. Aldehyde dehydrogenases: From eye crystallins to metabolic disease and cancer stem cells. Chemico-Biological Interactions. 2013;202(1-3):2-10. doi:10.1016/j.cbi.2012.10.026	cell culture			review
USA	Wang A, Leong DJ, He Z, Xu L, Liu L, Kim SJ, Hirsh DM, Hardin JA, Cobelli NJ, Sun HB. Procyanidins Mitigate Osteoarthritis Pathogenesis by, at Least in Part, Suppressing Vascular Endothelial Growth Factor Signaling. Int J Mol Sci. 2016 Dec 9;17(12):2065. doi: 10.3390/ijms17122065. PMID: 27941690; PMCID: PMC5187865.	animals		administration assay	
USA	Wei F, Neal CJ, Sakthivel TS, Kean T, Seal S, Coathup MJ. Multi-functional cerium oxide nanoparticles regulate inflammation and enhance osteogenesis. Materials Science & Engineering: C. 2021;124:N.PAG. doi:10.1016/j.msec.2021.112041	cell culture		administration assay	
USA	Wimalawansa SJ. Vitamin D Deficiency: Effects on Oxidative Stress, Epigenetics, Gene Regulation, and Aging. Biology (2079-7737). 2019;8(2):30. doi:10.3390/biology8020030				review
USA	Xia WF, Jung JU, Shun C, Xiong S, Xiong L, Shi XM, Mei L, Xiong WC. Swedish mutant APP suppresses osteoblast differentiation and causes osteoporotic deficit, which are ameliorated by N-acetyl-L-cysteine. J Bone Miner Res. 2013 Oct;28(10):2122-35. doi: 10.1002/jbmr.1954. PMID: 23649480; PMCID: PMC7104794.	animals		functional assay	
USA	Yousefzadeh, M. J., et al. "Fisetin is a Senotherapeutic that Extends Health and Lifespan." EBioMedicine, vol. 36, 2018, pp. 18-28. SCOPUS, www.scopus.com, doi:10.1016/j.ebiom.2018.09.015.		periodontitis	administration assay	
USA	Zigman, W. B. "Atypical Aging in Down Syndrome." Developmental Disabilities Research Reviews, vol. 18, no. 1, 2013, pp. 51-67. SCOPUS, www.scopus.com, doi:10.1002/ddrr.1128.				review

Table S3. The Joanna Briggs Institute Critical Appraisal Checklist for analytical cross-sectional studies.

Study	1. Were the criteria for inclusion in the sample clearly defined?	2. Were the study subjects and the setting described in detail?	3. Was the exposure measured in a valid and reliable way?	4. Were objective, standard criteria used for measurement of the condition?	5. Were confounding factors identified?	6. Were strategies to deal with confounding factors stated?	7. Were the outcomes measured in a valid and reliable way?	8. Was appropriate statistical analysis used?	Risk of bias
Botre et al. 2015.	Yes	Yes	Yes	Yes	Yes	Yes	Yes	Yes	Low
Deng et al. 2011.	Yes	Yes	Yes	Yes	Yes	Yes	Yes	Yes	Low
Mlakar et al. 2012.	Yes	Yes	Yes	Yes	Yes	Yes	Yes	Yes	Low
Yamada et al. 2003.	No	Yes	Yes	Yes	Yes	Yes	Yes	Yes	Low
Oh et al. 2007.	Yes	Yes	Yes	Yes	Yes	Yes	Yes	Yes	Low
Mlakar et al. 2012.	Yes	Yes	Yes	Yes	Yes	Yes	Yes	Yes	Low
Michaëlsson et al. 2021.	No	No	Yes	Yes	Yes	Yes	Yes	Yes	Low
Mlakar et al. 2011.	Yes	Yes	Yes	Yes	Yes	Yes	Yes	Yes	Low
Mlakar et al. 2010.	Yes	Yes	Yes	Yes	No	No	Yes	Yes	Low

Table S4. The Joanna Briggs Institute Critical Appraisal Checklist for case-control studies.

Study	1. Were the groups comparable other than the presence of disease in cases or the absence of disease in controls?	2. Were cases and controls matched appropriately?	3. Were the same criteria used for identification of cases and controls?	4. Was exposure measured in a standard, valid and reliable way?	5. Was exposure measured in the same way for cases and controls?	6. Were confounding factors identified?	7. Were strategies to deal with confounding factors stated?	8. Were outcomes assessed in a standard, valid and reliable way for cases and controls?	9. Was the exposure period of interest long enough to be meaningful?	10. Was appropriate statistical analysis used?	Risk of bias
Al-E-Ahmad et al. 2018.	Yes	Yes	Yes	Yes	Yes	Yes	Yes	Yes	Yes	Yes	Low
Usategui-Martín, et al. 2022.	Yes	Yes	Yes	No	Yes	Yes	Yes	Yes	Yes	Yes	Low
Senosi et al. 2022.	Yes	Yes	Yes	Yes	Yes	Yes	No	Yes	Yes	Yes	Low
Liu et al. 2009.	Yes	Unclear	Yes	Yes	Yes	Yes	Yes	Yes	Yes	Yes	Low

Table S5. The Joanna Briggs Institute Critical Appraisal Checklist for cohort studies

Study	1. Were the two groups similar and recruited from the same population?	2. Were the exposures measured similarly to assign people to both exposed and unexposed groups?	3. Was the exposure measured in a valid and reliable way?	4. Were confounding factors identified?	5. Were strategies to deal with confounding factors stated?	6. Were the groups/participants free of the outcome at the start of the study (or at the moment of exposure)?	7. Were the outcomes measured in a valid and reliable way?	8. Was the follow up time reported and sufficient to be long enough for outcomes to occur?	9. Was follow up complete, and if not, were the reasons to loss to follow up described and explored?	10. Were strategies to address incomplete follow up utilized?	11. Was appropriate statistical analysis used?	Risk of bias
Mlakar et al. 2012.	Yes	Yes	Yes	Yes	Yes	Yes	Yes	No	No	No	Yes	Low
